# Iron overload promotes myeloid differentiation of normal hematopoietic stem cells and educates macrophage mediated immunosuppression in acute myeloid leukemia

**DOI:** 10.3389/fimmu.2025.1626888

**Published:** 2025-08-13

**Authors:** Feifei Yang, Shulin Luo, Dan Yang, Xiaoxi Cui, Dongyue Zhang, Hao Wang, Yifei Li, Wanzhen Xie, Lina Wang, Xiuqun Zhang, Guoguang Zheng, Xuezhong Zhang

**Affiliations:** ^1^ Nanjing First Hospital, Nanjing Medical University, Nanjing, China; ^2^ State Key Laboratory of Experimental Hematology, National Clinical Research Center for Blood Diseases, Haihe Laboratory of Cell Ecosystem, Institute of Hematology & Blood Diseases Hospital, Chinese Academy of Medical Sciences & Peking Union Medical College, Tianjin, China; ^3^ Tianjin Institutes of Health Science, Tianjin, China

**Keywords:** acute myeloid leukemia, iron overload, myeloid differentiation, leukemia-associated macrophages, immunosuppression

## Abstract

**Background:**

The hematopoietic ecosystem comprises both cellular components such as hematopoietic stem cells (HSCs) and immune cells as well as non-cellular components including iron. Systemic iron overload, which leads to serious complications and affects both patients’ quality of life and overall survival, is a common clinical challenge in patients with acute myeloid leukemia (AML). We previously elucidated the direct effects of iron overload on AML cells. It’s worth noting that iron overload remodels the hematopoietic ecosystem. However, whether and how remodeled leukemic microenvironment with overloaded iron regulates normal HSCs and immune cells, especially leukemia-associated macrophages (LAMs), in AML have not been elucidated.

**Methods:**

The MLL-AF9-induced AML (MA9) cells were originated from c-kit^+^ BM cells enriched from C57BL/6J mice that infected with MSCV-MLL-AF9-GFP retrovirus. The MA9 AML mouse model was established by transplantation of MA9 cells into C57BL/6 mice. MA9 mice were *i.p.* administered with iron dextran every other day for a total of 6 times to established the iron overload MLL-AF9-induced AML mouse model (MA9/FE). HSC maintenance and differentiation was assessed by flow cytometry, cell proliferation, cell apoptosis, colony forming and competitive transplantation assays. LAM activation and function was analyzed by RNA-sequencing, flow cytometry and coculture assay. Intravenous clodronate liposome administration was employed to reduce LAMs in AML.

**Results:**

Iron overload skewed myeloid differentiation of normal HSCs. Furthermore, iron overload affected LAMs in the AML microenvironment by promoting LAM polarization toward an M2 phenotype. Functionally, iron overload decreased the phagocytic function of LAMs against leukemia cells and inhibited LAM-induced T cell activation by acquiring a tolerogenic phenotype with aberrant immune checkpoints. Moreover, depletion of LAMs attenuated iron overload caused acceleration of AML progression.

**Conclusions:**

Collectively, this study reveals the significance of iron overload in remodeling hematopoietic ecosystem and affecting HSC and LAM function in AML, providing new insights into the multifaceted role of iron overload in leukemia.

## Introduction

Iron is an essential element involved in numerous fundamental intracellular and extracellular physiologic processes. Iron homeostasis disorders play vital roles in the pathophysiology of various diseases as they cause diverse functional changes and progressive damages to organs (1–5). The relationship between the dysregulation of iron metabolism and the occurrence and progression of hematologic diseases has been reported (6). Acute myeloid leukemia (AML) is a hematologic malignancy characterized by the unlimited proliferation of myeloid precursor cells in the bone marrow (BM) with poor outcome although great progress has been achieved (7, 8). Iron overload reshapes the microenvironment, which has adverse effects on the initiation and progression of AML. Elevated iron promotes myelodysplastic syndromes (MDS)-AML transformation by causing genetic and chromosomal abnormalities (9), which could be attenuated by the treatment of iron chelators (10). Clinical studies have revealed that a high iron level is associated with a suppressed autologous stem cell mobilization, a poorer overall survival, and a higher incidence of relapse in AML patients (11–13). Our previous work demonstrated that the high level of iron correlated with a worse prognosis in AML patients and a shortened survival time in AML mice (14). Although we previously demonstrated that iron overload directly regulated AML cells by decreasing leukemic stem cells (LSCs) levels but increasing the tumor load in BM and extramedullary tissues via promoting the proliferation of leukemia cells through the upregulation of *FOS* (14), a comprehensive interpretation of the pathologic roles of iron overload in leukemia, especially the crosstalk between iron overload leukemic microenvironment and normal hematopoietic components, is less well-defined.

Leukemia cells highjack and destroy microenvironments, potentially shifting the equilibrium of microenvironments from a state that supports steady-state hematopoiesis to the conditions in favor of leukemogenesis and progression. Multiple types of cells including leukemia cells themselves (15, 16), normal hematopoietic cells (17, 18), immune cells (19), *etc.* are affected and contribute to the process. Hematopoietic stem cells (HSCs) are unique and hierarchically organized subpopulations of blood cells that maintain the hematopoietic system through self-renewal and generation of progenitor cells and mature cells of various lineages through differentiation in response to physiologic demands (20). Previous work demonstrated that leukemic microenvironment exhibited a great impact on HSCs as well as normal hematopoiesis (17, 18). However, iron overload further remodels the leukemic microenvironment. Evidence has suggested that iron may play important roles in hematopoiesis under steady state and stress conditions. Microbiota, antifungal agents, or transferrin receptor 1 (Tfr1)-mediated iron uptake have been shown to regulate HSC fate decisions in the BM (21–23). F-box and leucine-rich repeats protein 5 (FBXL5) or ferritin heavy chain 1 (*FTH1*)-mediated cellular iron homeostasis is required for the maintenance of HSCs functions (19, 24). In addition, the increased iron uptake by splenic HSCs has been shown to promote TET2-dependent erythroid regeneration in hemolytic anemia-induced stress erythropoiesis. However, how remodeled leukemic microenvironment with overloaded iron affect HSCs has not been elucidated.

The immunomicroenvironment constituted by mature immune cells and immune regulatory molecules participates in diverse physiologic and pathologic processes. Specifically, leukemia cells induce a leukemic immunomicroenvironment with suppressed immune response to leukemia cells, thereby impairing the anti-leukemia immunity, which in turn contributes to leukemia progression. Evidence demonstrated that a variety of immune cells including neutrophils (25), macrophages (26), NK cells (27, 28) and T cells (29), were reprogramed to have leukemia-associated characteristics and played pathologic roles. It is worth noting that leukemia-associated macrophages (LAMs) were educated by the leukemic microenvironment to exhibit unique phenotypes and functions with great heterogeneity in our previous studies (30–32), highlighting a context- and tissue-dependent macrophage polarization. However, the leukemic microenvironment becomes a highly dynamic structure remodeled by iron overload. It was reported that iron played important roles on the polarization and function of macrophages under distinct conditions, including *in vitro* cytokine stimulation and some *in vivo* disease models, such as infection, metabolic disorders, and solid cancers (33–38). Nevertheless, how remodeled leukemic microenvironment with overloaded iron reshapes LAMs has not been elucidated.

In this study, we investigated the impacts of the remodeled leukemic microenvironment with overloaded iron on HSCs and immune cells by using an iron overload MLL-AF9-induced mouse AML model. The results demonstrated that iron overload skewed myeloid differentiation of normal HSCs. Furthermore, iron overload promoted LAMs to exhibit a phenotype with increased M2-related characteristics, decreased phagocytotic potential and enhanced immunosuppression ability. Moreover, depletion of LAMs attenuated iron overload-caused acceleration of AML progression. Therefore, our findings provide new insights into the mechanisms how iron overload exerts adverse effects in AML and broaden our knowledge of the multifaceted roles of iron overload in malignancies.

## Materials and methods

### Gene expression datasets

Public datasets of leukemia patients were downloaded from The Cancer Genome Atlas (TCGA). The correlation between gene expressions of *FTH1* or *FTL* and *CD68* or *CD163* in AML patients was studied. The detailed characteristics of the patients accessible from the database are summarized in **Supplementary Table S1**.

### Mice

Six to eight week-old C57BL/6J (CD45.2) and B6.SJL (CD45.1) mice were provided by the Animal Centre of the Institute of Hematology and Blood Diseases Hospital, CAMS & PUMC. Mice were maintained in SPF-certified facilities, and all procedures for animal experiments were approved by the Animal Care and Use Committees at the Institution.

### AML mouse models

The establishment of MLL-AF9-induced AML mouse model (MA9) was previously described (17, 39). Briefly, c-kit^+^ BM cells were enriched from C57BL/6J mice and infected with MSCV-MLL-AF9-GFP retrovirus before transplantation into C57BL/6 mice.

We recently established the iron overload MLL-AF9-induced AML mouse model (abbreviated as MA9/FE) (14). Briefly, GFP^+^ AML cells were isolated from MA9 mice and transplanted into C57BL/6 mice. One week after transplantation, the recipient AML mice were *i.p.* administered with 0.2 mL iron dextran (Pharmacosmos A/S, Denmark) at a concentration of 25 mg/mL every other day for a total of 6 times. An equal volume of phosphate-buffered saline (PBS) was administrated to the control AML mice (also abbreviated as MA9). C57BL/6 mice without AML cell transplantation were *i.p.* administered with an equal volume of iron dextran or PBS (abbreviated as WT/FE or WT).

### Cell culture

All cells were cultured in RPMI-1640 supplemented with 5% fetal bovine serum (FBS) and antibiotics in a humidified atmosphere of 5% CO_2_ at 37°C. All culture supplies were endotoxin free.

### Antibodies

Fluorescence-conjugated antibodies against F4/80 (APC) and Ki67 (PE) and were purchased from BD Biosciences (USA). Fluorescence-conjugated antibodies against CD45.2 (PE), CD45.2 (PerCP-Cy5.5), Sca-1 (APC), CD117/c-Kit (PE-Cy7), CD16/32 (PerCP-Cy5.5), CD34 (BV421), Flt3 (PE), CD3 (PerCP-Cy5.5), CD4 (PE-Cy7), CD8 (APC-Cy7), CD19 (PE), CD25 (FITC), CD44 (APC), Mac-1/CD11b (PE-Cy7), Gr-1 (PE-Cy7), Gr-1 (Pacific blue), CD115 (BV711), CD115 (PE), CD47 (APC), SIRPα(APC-Cy7), PD-L1 (PE-Cy7), PD-1 (BV421), CD200 (PE-Cy7), CD200R (FITC), Galectin-9 (PerCP-Cy5.5), Tim-3 (PerCP-Cy5.5) and Annexin V (PE) were purchased from Biolegend (USA).

### Flow cytometric analysis and cell sorting

EasySep™ Mouse Hematopoietic Progenitor Cell Isolation Kit (#19856, Stem Cell Technologies, USA) was used to enrich the Lin^-^ cells from the mouse BM. Lin^-^ cells were enriched by using streptavidin-coated magnetic particles and biotinylated antibodies (CD5, CD11b, CD19, CD45R/B220, Gr-1 and Ter119, 7-4; APC-Cy7 conjugated) to remove non-hematopoietic stem/progenitor cells (HSPCs). Then, LSK^+^ cells were gated as Lin^-^Sca1^+^c-Kit^+^ from the GFP^-^ population. Antibodies against CD34 and Flt3 were used to identify CD34^-^Flt3^−^ LT-HSC, CD34^+^Flt3^-^ ST-HSC and CD34^+^Flt3^+^ MPP from the GFP^-^Lin^-^Sca1^+^c-Kit^+^ population. CD34 and CD16/32 antibodies were used to identify CD34^+^CD16/32^+^ GMP, CD34^+^CD16/32^-^ CMP and CD34^-^CD16/32^-^ MEP from the GFP^-^Lin^-^Sca1^-^c-Kit^+^ population. These HSPCs were used for further analysis. The BM macrophages were gated as the Gr-1^lo^F4/80^+^CD115^int^SSC^int/lo^ subpopulation from the GFP^-^ population. Canto II and LSR II flow cytometers (BD Biosciences) were used for FACS analysis. FACS Aria III (BD Biosciences) was used for cell sorting. Data analysis was carried out using FlowJo software (version 7.6.1).

### Cell proliferation assay

Ki67 staining assays were used to detect cell proliferation of primary LSKs. Normal LSKs from MA9 and MA9/FE mice were fixed and permeabilized by Cytofix/Cytoperm™ Fixation/Permeabilization Solution Kit (BD Biosciences, San Jose, CA) according to the manufacturer’s protocols. Then, cells were stained with an anti-Ki67-PE antibody, followed by Hoechst 33342 before FACS analysis.

### Cell apoptosis analysis

Annexin V/PI staining assay was used to detect cell apoptosis of primary LSKs. Normal LSKs from MA9 and MA9/FE mice were prepared and washed twice with pre-cold PBS buffer. Then, cells were incubated with an Annexin V-PE antibody, followed by PI before FACS analysis.

### Colony forming assay

Freshly sorted normal LSKs from MA9 and MA9/FE mice were suspended in M3434 complete medium (Stem Cell Technologies, USA) and seeded in 24-well plate at 500 cells/500 μl/well and cultured according to the manufacturer’s instructions. The number of burst-forming unit erythroid (BFU-E), colony-forming unit granulocyte-macrophage (CFU-GM), and colony-forming unit granulocyte, erythroid, macrophage, and megakaryocyte (CFU-GEMM) was counted on day 8.

### Transplantation assays

For competitive LSK transplantation, normal LSKs (CD45.2, 1×10^3^) from MA9 or MA9/FE mice and WT competitor cells (whole bone marrow cells (WBMCs)) (CD45.1, 5×10^5^) were transplanted into lethally irradiated (9.5 Gy) recipient B6.SJL mice (CD45.1). The reconstitution of PB cellularity was analyzed by FACS every four weeks post-transplantation. Sixteen weeks after transplantation, BM cells from the recipient mice were collected, and the chimera rate of the HSPC compartment and lineage contribution was evaluated by FACS.

### Latex bead uptake experiments

Latex bead uptake experiments were used to measure the phagocytic activity of macrophages. Prior to FACS analysis, BM macrophages from WT, WT/FE, MA9 or MA9/FE mice were incubated with FITC-labeled 2 µm latex beads (Sigma-Aldrich, USA) for 15 mins.

### Phagocytotic activity of macrophages against leukemia cells

Coculture assays were used to test the phagocytosis of leukemia cells by macrophages. BM macrophages from WT, WT/FE, MA9 or MA9/FE mice were cocultured with GFP^+^ BM AML cells at the ratio of 1:2 in 24-well plates for 18 hrs. Leukemia cells cultured alone were used as blank controls. After 18 hrs, total cells were counted by hemocytometer and the proportion of GFP^+^ cells was analyzed by FACS (18 hrs (test)). The phagocytotic rate was calculated as follows: (100%- test) - (100%- blank control test).

### Regulatory effect of macrophages on T cell activation

Co-culture assays were used to test the regulatory effects of macrophages on T cells. BM macrophages from WT, WT/FE, MA9 or MA9/FE mice were co-cultured for 48 hrs with T cells that were sorted from the spleen of normal mice, at the ratio of 1:1 in 48-well plates in the presence of Dynabeads Mouse T-Activator CD3/CD28 (Gibco, USA). Then, the cells were harvested and resuspended in 100 μl PBS. After staining with antibodies at 4°C and washing with PBS, the cells were analyzed by FACS.

### Quantitative real-time PCR

Cells were lysed and total RNA was isolated using RNeasy Mini Kit (Qiagen, Valencia, CA). Reverse transcription was achieved using Transcript All-in-one First-Strand cDNA Synthesis SuperMix (TransGen Biotech, China). qRT-PCR was performed on 0.1-QuantStudio 5 and 0.2-QuantStudio 5 (Thermo Fisher Scientific, USA). The expression level of each gene was normalized to the expression level of GAPDH. Sequences for primers are listed in **Supplementary Table S2**.

### RNA-seq

BM macrophages were sorted from WT, WT/FE, MA9 and MA9/FE mice by FACS. An RNA sequencing library was prepared and sequenced using an Illumina Nova Seq 6000 by the Beijing Novogene following standard protocols. The data was analyzed by Beijing Novogene and is available in the NCBI’s Gene Expression Omnibus under the accession number GSE226727.

### Two-dimensional illustration of macrophage phenotypes

A two-dimensional illustration was established to describe the activation phenotype of macrophages (31). For each gene, the value of macrophage from WT BM was designated as 0. The M1 and M2 values of macrophages were calculated and the mean values of relative expression of genes were used for calculation:

M1 value=∑max(-ΔΔCtM1,0) - ∑min(-ΔΔCtM2,0);
M2 value=∑max(-ΔΔCtM2,0) - ∑min(-ΔΔCtM1,0).

### Statistical analysis

The statistical analyses were performed using the GraphPad Prism 6.0 software (San Diego, CA) and the SPSS17.0 software package (SPSS, Chicago, IL). All data were expressed as the mean ± SEM and statistical significance was calculated using an unpaired Student’s *t*-test (two groups) or one-way ANOVA (≥ three groups). Kaplan–Meier analysis was employed to compare the survival time. *P <*0.05 was considered statistically significant.

## Results

### Iron overload affects normal HSC functions in AML mice

**Figure 1:**
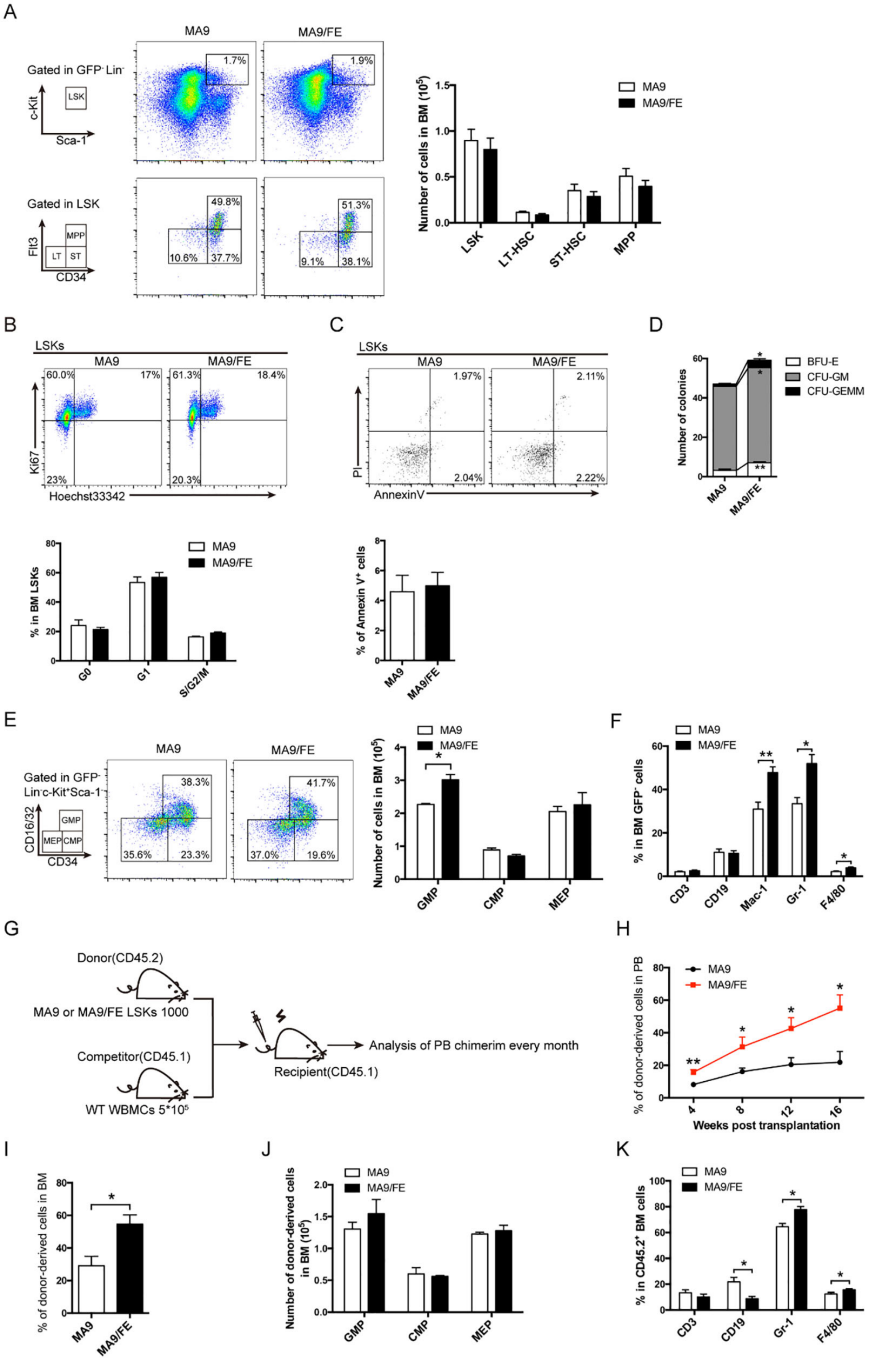
Normal HSPCs and more mature cells of multiple lineages in iron-overload AML mice. **(A)** Representative FACS plots and frequencies of normal HSC subpopulations (LSK: Lin^-^c-Kit^+^Sca1^+^, LT-HSC: CD34^-^Flt3^−^LSK^+^ ST-HSC: CD34^+^Flt3^-^LSK^+^, MPP: CD34^+^Flt3^+^LSK^+^) in the BM of MA9 and MA9/FE mice (n = 5). **(B)** Normal HSCs from the BM of MA9 and MA9/FE mice were stained with Ki67 and Hoechst 33342. The gating strategy for G0 (bottom left), G1 (top left), and S/G2/M (top right) is shown (top panel), and the percentage is plotted (n=5) (bottom panel). **(C)** Normal HSCs from the BM of MA9 and MA9/FE mice were stained with Annexin V and PI. The gating strategy for early (bottom right) and late (top right) phase apoptotic cells is shown (top panel), and the percentage of is plotted (n=5) (bottom panel). **(D)** Normal HSCs from the BM of MA9 or MA9/FE mice were seeded onto 24-well plates (500 cells/well) for colony forming assays, and the numbers of BFU-E, CFU-GM, and CFU-GEMM are plotted. **(E)** Representative FACS plots and frequencies of HPC subpopulations (GMP: CD34^+^CD16/32^+^, CMP: CD34^+^ CD16/32^−^, MEP: CD34^-^ CD16/32^-^, gated from GFP^-^Lin^-^Sca1^-^c-Kit^+^ population) in the BM of MA9 and MA9/FE mice (n = 5). **(F)** Lineage distribution in BM assessed by FACS (n = 5). **(G-K)** Competitive BM transplantation was performed and mice were sacrificed 16 weeks post-transplantation for chimerism analysis (n = 6). The schematic of the experiment design **(G)**, the chimerism of PB CD45.2^+^ cells at indicated time points **(H)** as well as the chimerism of BM CD45.2^+^ cells **(I)**, the absolute numbers of CD45.2^+^ HPC populations **(J)** and the percentage of CD45.2^+^ multiple lineages **(K)** are shown. The results are from three independent experiments. Unpaired Student’s t test; *p < 0.05; **p < 0.01.

To determine the impacts of iron overload on normal HSCs in AML, the iron overloaded MLL-AF9-induced AML mouse model was successfully established and iron overload was validated (**Supplementary Figure S1**). The frequency of GFP^-^Lin^-^c-Kit^+^Sca1^+^ cells (normal LSK) in the BM of MA9 and MA9/FE mice was analyzed, and no statistical difference was detected between the two groups (**Figure 1A**). Then, CD34 and Flt3 were used to identify the HSC subpopulations, *i.e.* CD34^-^Flt3^−^ LT-HSC, CD34^+^Flt3^-^ ST-HSC and CD34^+^Flt3^+^ MPP. No significant difference was observed for those subpopulations between two groups (**Figure 1A**). These results suggest that iron overload does not influence the frequency of normal HSCs and HSC subpopulations in AML.

Next, we investigated whether iron overload had an impact on the proliferation and apoptosis of normal HSCs in AML. No significant difference was observed between MA9 and MA9/FE mice (**Figures 1B, C**). Colony forming ability determines the self-renewal potential of HSCs and partly reflects HSC function. MA9/FE HSCs formed significantly more BFU-E, CFU-GM and CFU-GEMM colonies than MA9 HSCs (**Figure 1D**, **Supplementary Figure S2A**). These results suggest that iron overload promotes the colony-forming capacity of normal HSCs in AML mice.

### Iron overload skews myeloid differentiation of normal HSCs in AML mice

To determine whether iron overload affects differentiation of HSCs, the frequency of normal HPCs and more mature cells of multiple lineages in the BM of MA9 and MA9/FE mice was evaluated. CD34 and CD16/32 were used to identify the committed progenitor cells, CD34^+^ CD16/32^+^ GMP, CD34^+^ CD16/32^−^ CMP, and CD34^-^ CD16/32^-^ MEP were based on the GFP^-^Lin^-^Sca1^-^c-Kit^+^ population. A significant increase in the frequency of GMP was observed in MA9/FE mice when compared with that in MA9 mice (**Figure 1E**). In addition, iron overload resulted in a prominent increase in more mature compartments of myeloid cells, such as Mac-1^+^, Gr-1^+^ and F4/80^+^ cells, in the BM (**Figure 1F**), suggesting that iron overload enhances myeloid lineage differentiation in AML.

Next, we performed a competitive repopulation assay to dissect the effect of iron overload on the hematopoietic reconstitution capacity of normal HSCs (**Figure 1G**). Donor-derived PB chimera rate of normal HSCs from MA9/FE mice was significantly higher than that from MA9 mice during the 16 weeks post-transplantation (**Figure 1H**, **Supplementary Figure S2B**). Moreover, similar results were also obtained for donor-derived BM chimera rate 16 weeks after transplantation (**Figure 1I**, **Supplementary Figure S2C**). These results indicate that iron overload promotes the hematopoietic reconstitution capacity of normal HSCs in AML. Though the donor-derived BM chimerism of HPCs populations, including CMP, GMP and MEP, showed no significant difference between MA9 and MA9/FE mice (**Figure 1J**, **Supplementary Figure S2D**), iron overload increased the donor-derived BM chimerism of myeloid lineages, including Gr-1^+^ and F4/80^+^ cells, while decreased the BM chimerism of CD19^+^ B cells (**Figure 1K**). Together, these findings indicate that iron overload enhances the capacity of hematopoietic reconstitution and skews the myeloid differentiation of normal HSCs in AML.

### More M2 macrophages correlates with a worse prognosis in iron overloaded AML patients

**Figure 2:**
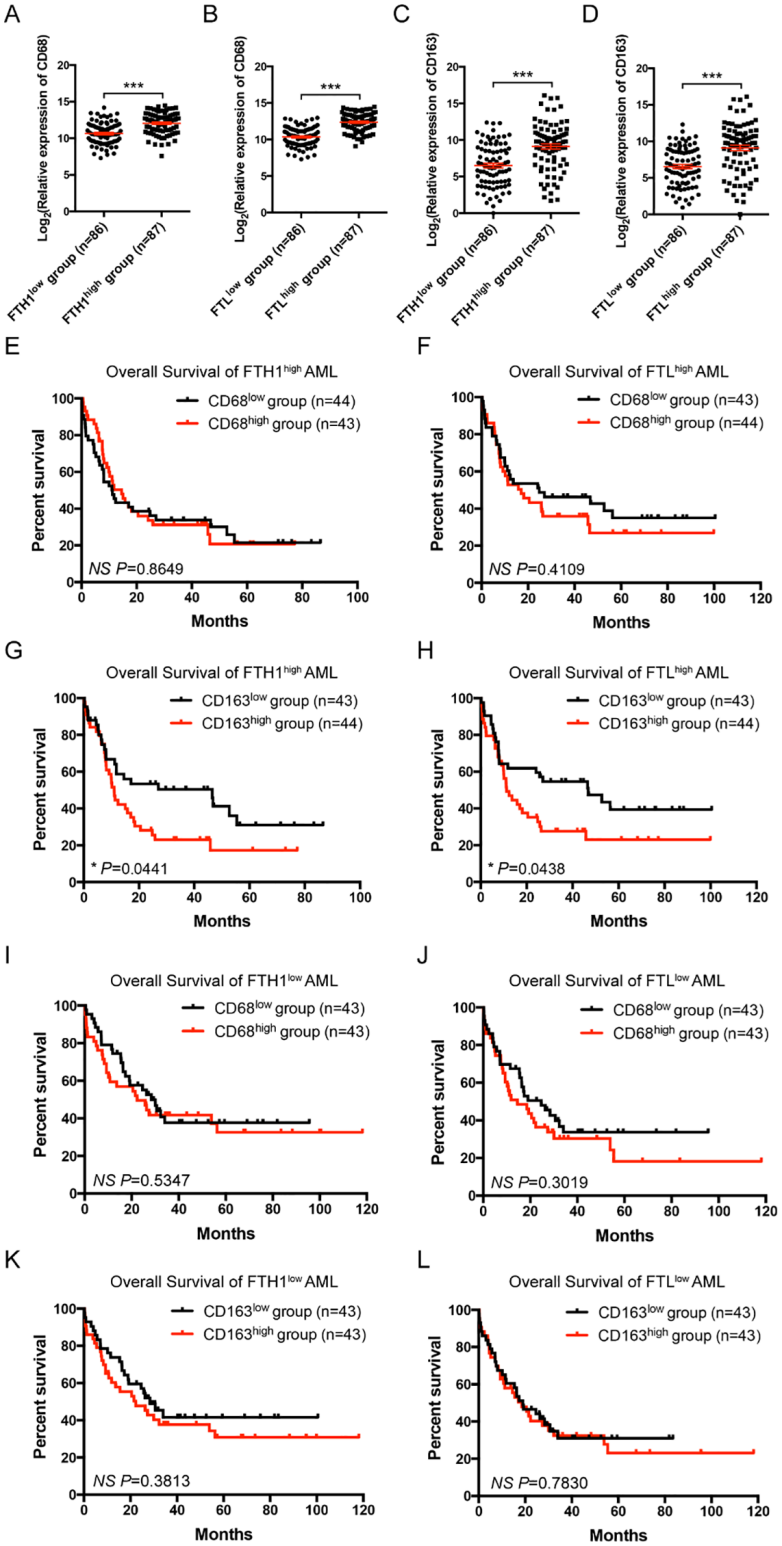
Correlation and clinical significance of ferritin and macrophage markers in AML patients. **(A-D)** Correlations between the relative expression of CD68 or CD163 and *FTH1* or *FTL* in TCGA are shown. AML cases were divided into the FTH1^high^, FTH1^low^, FTL^high^, and FTL^low^ groups, based on the median gene expression value of *FTH1* or *FTL*, respectively, and the relative expression of CD68 **(A, B)** or CD163 **(C, D)** is plotted. **(E-H)** In the FTH1^high^ or FTL^high^ groups, AML cases were divided into CD68^high^, CD68^low^, CD163^high^, and CD163^low^ subgroups, based on the median gene expression value of CD68 or CD163, respectively, and the OS of AML patients was compared by Kaplan-Meier analysis. **(I-L)** In the FTH1^low^ or FTL^low^ groups, AML cases were divided into CD68^high^, CD68^low^, CD163^high^, and CD163^low^ subgroups, based on the median gene expression value of CD68 or CD163, respectively, and the OS of AML patients was compared by Kaplan-Meier analysis. Unpaired Student’s t test; *p < 0.05; ***p < 0.001..

Ferritin consists of heavy and light chains, encoded by *FTH1* and *FTL* genes. CD68 and CD163 are typical markers for macrophages and M2 macrophages in humans. To assess the clinical significance of iron overload and macrophages in AML, the public TCGA database was used to analyze the expression levels of *FTH1*/*FTL* and *CD68/CD163* in leukemia patients and their correlations with disease prognosis. AML patients from TCGA datasets were divided into the FTH1^high^, FTH1^low^, FTL^high^, and FTL^low^ groups, based on the median gene expression value of *FTH1* or *FTL*, respectively. FTH1^high^ and FTL^high^ groups expressed higher level of CD68 or CD163 than their respectively counterparts, while FTH1^low^ and FTL^low^ groups expressed lower level of CD68 or CD163 than their respectively counterparts (**Figures 2A–D**). We previously demonstrated that FTH1^high^ group had a worse prognosis than FTH1^low^ group (14). We further analyzed the overall survival (OS) of the CD68^high^, CD68^low^, CD163^high^ and CD163^low^ subgroups in the FTH1^high^ or FTL^high^ groups. The results showed that no significant difference was detected between the CD68^high^ and CD68^low^ subgroups (**Figures 2E, F**) whereas the OS in the CD163^high^ subgroup was significantly shorter than that in the CD163^low^ subgroup (**Figures 2G, H**). The OS of the CD68^high^, CD68^low^, CD163^high^ and CD163^low^ subgroups in the FTH1^low^ or FTL^low^ groups was also analyzed showing that no significant difference was detected between the CD68^high^ and CD68^low^ subgroups (**Figures 2I, J**), and between the CD163^high^ and CD163^low^ subgroup (**Figures 2K, L**). These results suggest that iron overload correlates with increased level of M2 macrophages and more M2 macrophages further correlates with a poor prognosis in iron overloaded AML patients.

### Iron overload educates LAM with more M2 characteristics

**Figure 3:**
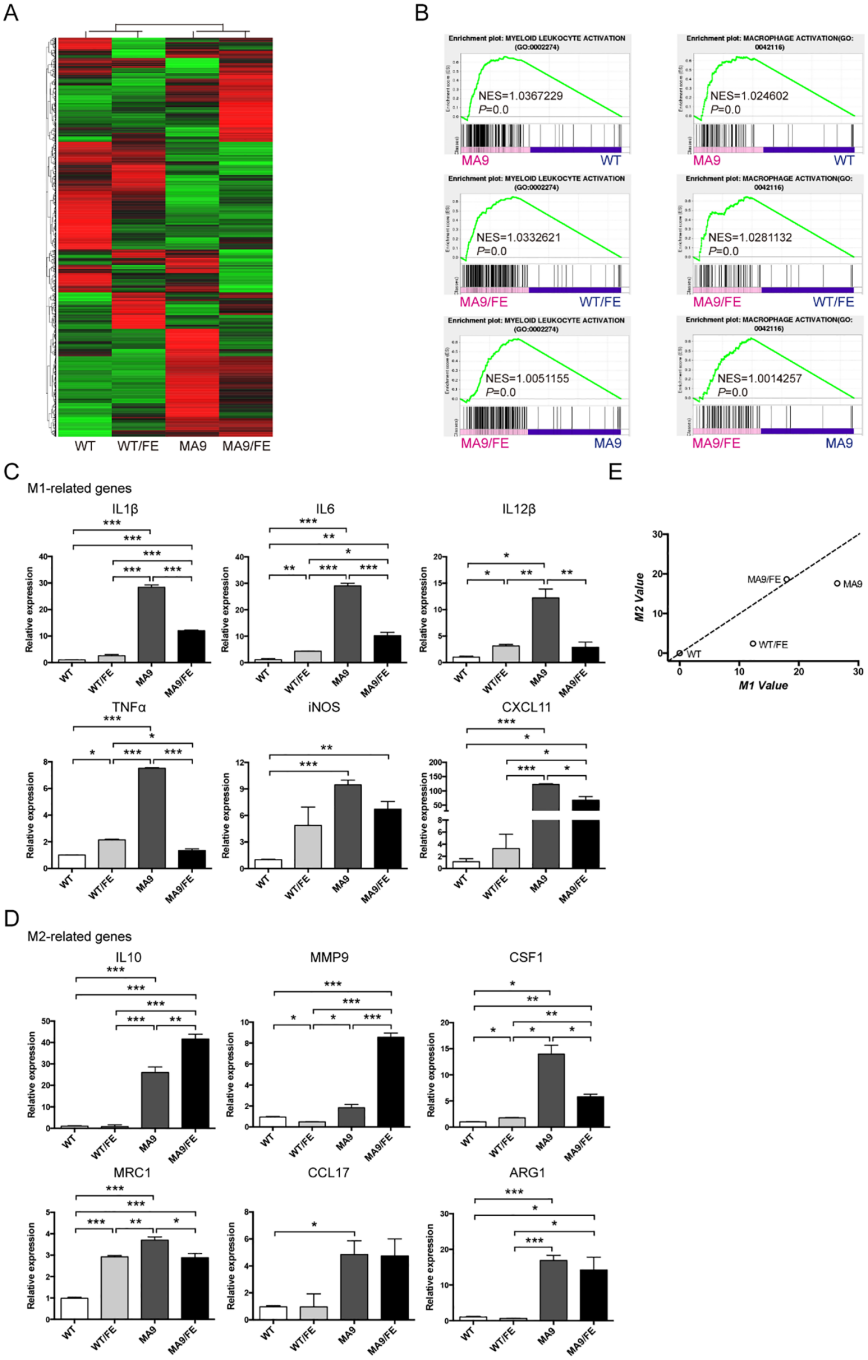
Iron overload affects the gene expression profile and activation phenotype of LAMs. Macrophages were sorted from the BM of MA9, MA9/FE mice and their respective normal counterparts (WT and WT/FE mice), and RNA-seq was performed. **(A)** DEG (FC ≥ 2, FDR ≤ 0.01) expression profiles are shown. **(B)** GSEA shows the enrichment of genes in annotations between MA9/FE LAMs and WT/FE macrophages or MA9 LAMs. Expression of M1 **(C)** and M2 **(D)** phenotype-associated genes in macrophages was detected by real-time PCR. For each gene, the RQ value of WT macrophages was designated 1.000, respectively. **(E)** A two-dimensional illustration of the macrophage phenotype is shown. For each gene, the value of the WT macrophages was designated 0. The mean values of the relative expression of genes mentioned above were used for the calculation and the M1 and M2 values. The results are from three independent experiments. Unpaired Student’s t test; *p < 0.05; **p < 0.01; ***p < 0.001..

The gene expression profile and activation phenotype of LAMs in the iron-overload AML microenvironment were studied. RNA-seq was performed to study gene expression profiles in the BM LAMs. Differentially expressed genes (DEGs) are plotted in **Figure 3A** (DEGs, FC ≥ 2, FDR ≤0.01), which reflect the modulatory effects of iron overload on LAM gene expression profile in leukemic microenvironment. A Venn diagram shows the numbers of DEGs between different pairs of samples (**Supplementary Figure S3**), and 29-shared DEGs are listed in **Supplementary Table S3**. Gene set enrichment analysis (GSEA) showed that the genes in the annotations, “myeloid leukocyte activation” and “macrophage activation”, were enriched in MA9/FE LAMs versus WT/FE macrophages or MA9 LAMs (**Figure 3B**). To study the activation phenotype of MA9/FE LAMs, the dynamic expression of phenotype-associated genes (6 genes for M1 and 6 genes for M2) was monitored. The expression levels of M1- and M2-related genes (**Figures 3C, D**) showed considerable differences among different samples. To obtain an intuitive view of the phenotypes of macrophages from different samples, a two-dimensional illustration of macrophage phenotypes was used. When WT macrophages were used as control, WT/FE macrophages and MA9 LAMs were in the lower area, *i.e.* with more M1 characteristics, whereas MA9/FE LAMs were in the upper area, *i.e.* with more M2 characteristics (**Figure 3E**). Interestingly, iron overload educated WT macrophages towards M1 phenotype (WT/FE macrophages) whereas educated MA9 LAMs towards M2 phenotype (MA9/FE LAMs) (**Figure 3E**). These results suggest that LAMs show distinct transcriptional modulations in response to iron overload in the leukemic environment, and exhibit increased M2-related characteristics, potentially resulting in different biological activities and functions.

### Iron overload decreases the phagocytic ability of LAMs

**Figure 4:**
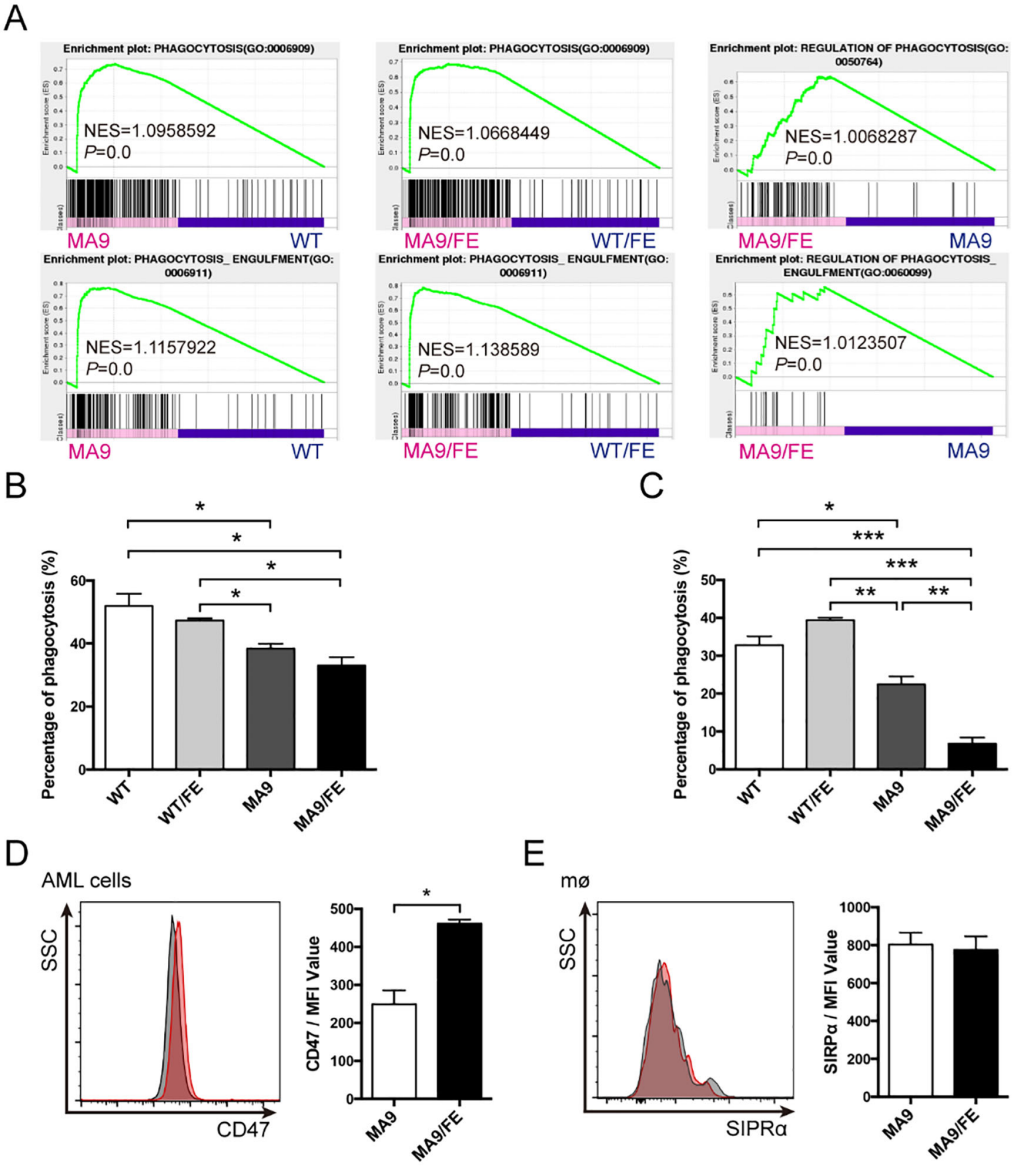
Iron overload decreases the phagocytic ability of LAMs. **(A)** GSEA shows the enrichment of genes in annotations between MA9/FE LAMs and WT/FE macrophages or MA9 LAMs. **(B)** Macrophages sorted from the BM of WT, WT/FE, MA9 or MA9/FE mice were cultured with FITC-labeled 2 μm latex beads for 15 mins, and the phagocytic activity was assessed by FACS (n=5). **(C)** Macrophages sorted from the BM of WT, WT/FE, MA9 or MA9/FE mice were cocultured with sorted leukemia cells for 18 hrs. Cells were collected, and the proportion of GFP^+^ cells was analyzed by FACS (n=5). AML cells cultured alone were used as a blank control. The phagocytotic rate was calculated as follows: (100%- 18 hrs (test))-(100%- blank control). **(D, E)** The expressions of CD47 on leukemia cells **(D)** and SIRPα on LAMs **(E)** were detected by FACS. The results are from three independent experiments. Unpaired Student’s t test; *p < 0.05; **p < 0.01; ***p < 0.001.

To evaluate the potential effects of iron overload on the functions of LAMs, DEG profiles were analyzed. GSEA showed that the genes in the annotations, “phagocytosis” and “phagocytosis engulfment”, were enriched in MA9 LAMs versus WT controls, MA9/FE LAMs versus WT/FE controls, and MA9/FE LAMs versus MA9 LAMs (**Figure 4A**). Phagocytosis is an important functional characteristic of macrophages. Hence, non-specific, and specific phagocytosis were assessed. Non-specific phagocytosis was assessed using the latex bead uptake experiments. MA9 and MA9/FE LAMs had a decreased phagocytotic ability, when compared with their respective control macrophages (**Figure 4B**, **Supplementary Figure S4A**). Specific phagocytosis of AML cells was assessed using *in vitro* co-culture experiments. We found that in addition to the decreased phagocytotic ability of MA9 and MA9/FE LAMs, the phagocytotic ability of MA9/FE LAMs was much lower than that of MA9 LAMs (**Figure 4C**, **Supplementary Figure S4B**).

CD47/SIRPα axis is a major pathway in repressing the activation and phagocytosis of macrophages. Thus, CD47 expression on leukemia cells and SIRPα expression on LAMs were investigated. CD47 expression was upregulated in MA9/FE AML cells (**Figure 4D**), whereas no difference was observed in SIRPα expression between MA9 and MA9/FE LAMs (**Figure 4E**). These results demonstrate that LAMs have a low phagocytotic potential against AML cells in response to the iron overload in the leukemic microenvironment.

### Iron overload promotes LAMs-mediated immunosuppression

**Figure 5:**
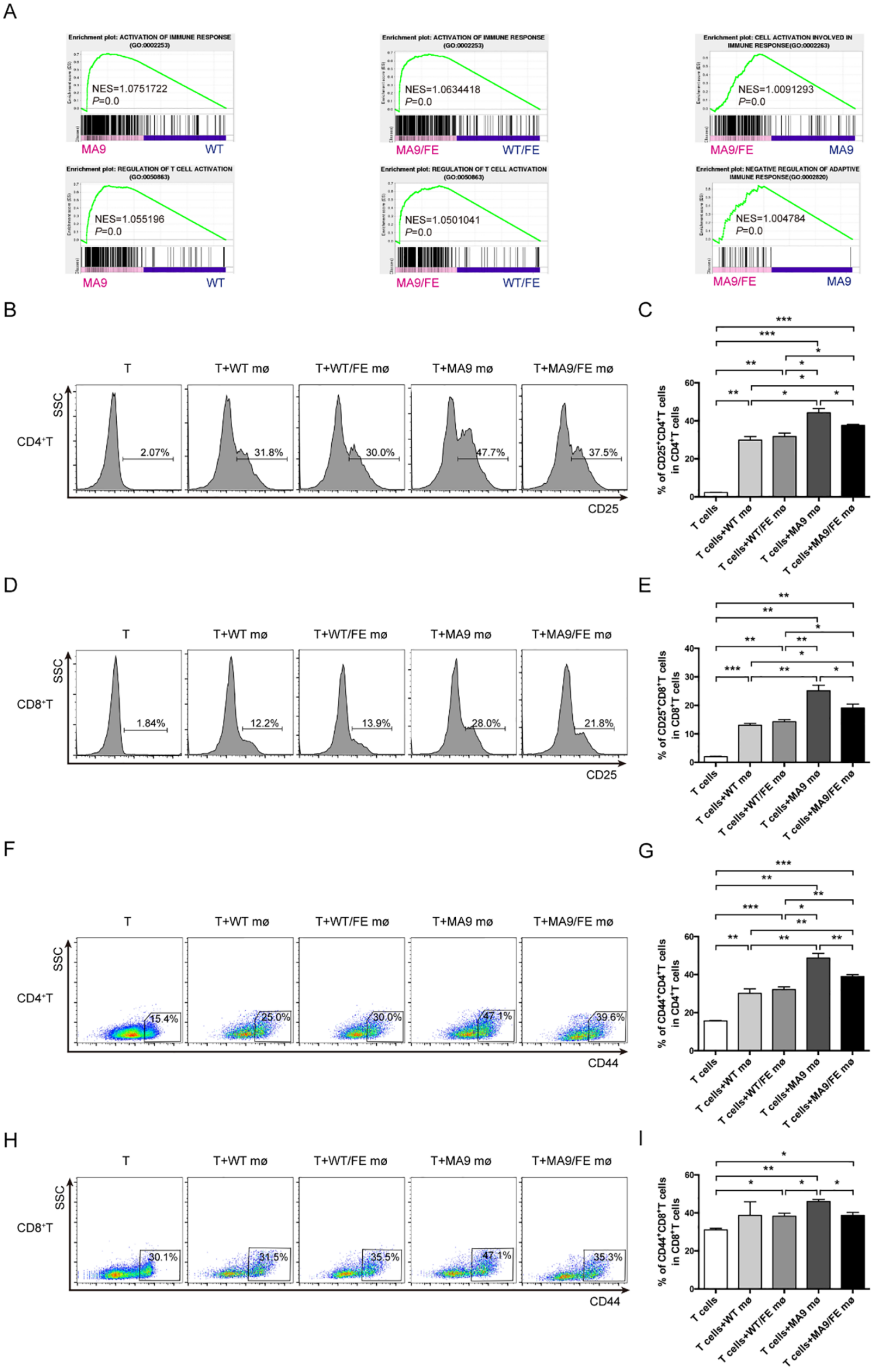
Iron overload promotes LAMs-mediated T cell activation inhibition. **(A)** GSEA shows the enrichment of genes in annotations between MA9/FE LAMs and WT/FE macrophages or MA9 LAMs. **(B-I)** Freshly sorted T cells from the spleen of WT mice were cocultured with macrophages from the BM of WT, WT/FE, MA9 or MA9/FE mice in 48-well plates for 48 hrs (n=5). T cells cultured without macrophages were set as the blank group. Typical FACS results **(B)** and the expression of CD25 in CD4^+^ T cells **(C)** are shown. Typical FACS results **(D)** and the expression of CD25 in CD8^+^ T cells **(E)** are shown. Typical FACS results **(F)** and the expression of CD44 in CD4^+^ T cells **(G)** are shown. Typical FACS results **(H)** and the expression of CD44 in CD8^+^ T cells **(I)** are shown. The results are from three independent experiments. Unpaired Student’s t test; *p < 0.05; **p < 0.01; ***p < 0.001.

GSEA results showed that the genes in the annotations, “activation of immune response” and “regulation of T cell activation”, were enriched in MA9 LAMs versus WT controls, and MA9/FE LAMs versus WT/FE controls. Furthermore, the annotations, “cells activation involved in immune response” and “negative regulation of adaptive immune response”, were enriched in MA9/FE LAMs versus MA9 LAMs (**Figure 5A**). Hence, the immunoregulatory potential of LAMs on T cells was analyzed using *in vitro* co-culture experiments. CD25 and CD44 expressions, which are generally related to T cell activation, were analyzed. A higher level of CD25 was detected in both CD4^+^ and CD8^+^ T cells co-cultured with MA9 or MA9/FE LAMs, than those co-cultured with their respective control macrophages. However, CD25 expression level decreased in CD4^+^ and CD8^+^ T cells co-cultured with MA9/FE LAMs, when compared with that with MA9 LAMs (**Figures 5B–E**). Furthermore, a higher level of CD44 was detected in CD4^+^ T cells co-cultured with MA9 or MA9/FE LAMs, than those co-cultured with their control macrophages. However, CD44 expression level decreased in CD4^+^ and CD8^+^ T cells co-cultured with MA9/FE LAMs, when compared with that with MA9 LAMs (**Figures 5F–I**). These results suggest that iron overload induces the inhibition of LAMs-mediated T cell activation.

### Iron overload promotes LAMs to acquire tolerogenic phenotypes that may contribute to T cell exhaustion

**Figure 6:**
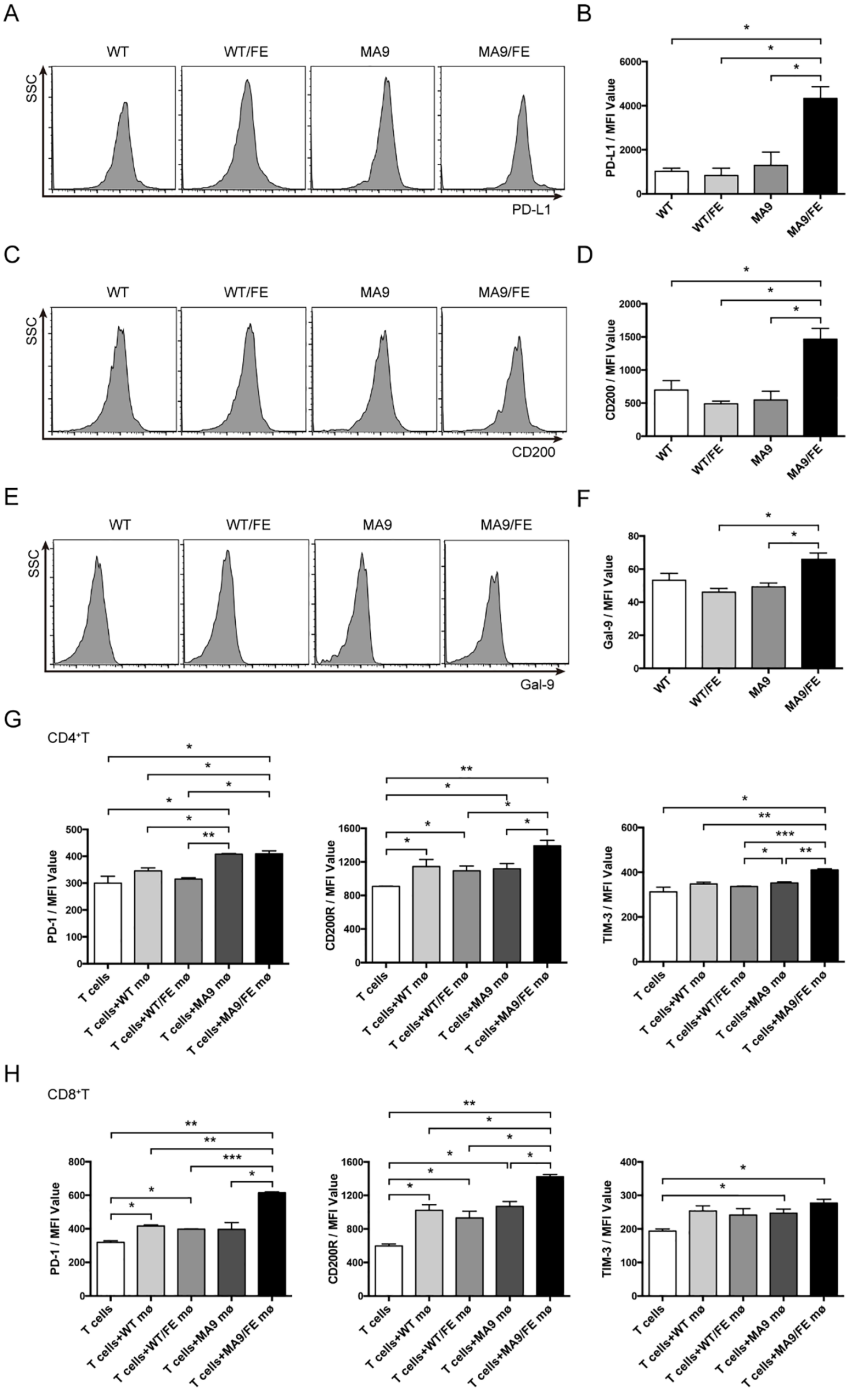
Iron overload promotes LAMs-mediated T cell exhaustion. Macrophages from the BM of WT, WT/FE, MA9 or MA9/FE mice were sorted, and the expression of immune checkpoint genes was analyzed. Typical FACS results **(A)** and the expression of PD-L1 in BM macrophages in MFI **(B)** are shown. Typical FACS results **(C)** and the expression of CD200 in BM macrophages in MFI **(D)** are shown. Typical FACS results **(E)** and the expression of Gal-9 in BM macrophages in MFI **(F)** are shown. **(G, H)** Freshly sorted T cells from the spleen of WT mice were cocultured with macrophages from the BM of WT, WT/FE, MA9 or MA9/FE mice in 48-well plates for 48 hrs (n=5). T cells cultured without macrophages were set as the blank group. The expressions of PD-1, CD200R and TIM-3 in CD4^+^ **(G)** or CD8^+^ **(H)** T cells were detected by FACS and are shown in MFI. The results are from three independent experiments. Unpaired Student’s t test; *p < 0.05; **p < 0.01; ***p < 0.001.

To further evaluate the potential mechanism of LAMs-induced immune dysfunction in iron-overload leukemic microenvironment, the expression of immune checkpoints on LAMs was detected. No difference was observed in the expression levels of PD-L1, CD200 and Galectin9, the immune checkpoint ligands, between MA9 LAMs and their control macrophages. However, MA9/FE LAMs expressed higher levels of these molecules than those on control macrophages or MA9 LAMs (**Figures 6A–F**).

Next, the expression levels of PD-1, CD200R and Tim3, the immune checkpoint receptors, on T cells after *in vitro* co-cultured with macrophages were detected. CD4^+^ T cells co-cultured with MA9 or MA9/FE LAMs expressed higher level of PD-1 than those co-cultured with control macrophages. CD4^+^ T cells co-cultured with MA9/FE LAMs expressed higher levels of CD200R and Tim3 than those co-cultured with control macrophages. Furthermore, CD4^+^ T cells co-cultured with MA9/FE LAMs had higher expression levels of CD200R and Tim3 than those co-cultured with MA9 LAMs (**Figure 6G**, **Supplementary Figures S5A–C**). CD8^+^ T cells co-cultured with MA9/FE LAMs expressed higher levels of PD-1 and CD200R than those co-cultured with control macrophages. Additionally, CD8^+^ T cells co-cultured with MA9/FE LAMs had higher expression levels of PD-1 and CD200R than those co-cultured with MA9 LAMs (**Figure 6H**, **Supplementary Figures S5D–F**). These results suggest that iron overload promotes LAMs acquisition of tolerogenic phenotypes that may contribute to T cell exhaustion.

### Iron overload accelerates AML progression and depletion of LAMs hinders AML progression

**Figure 7:**
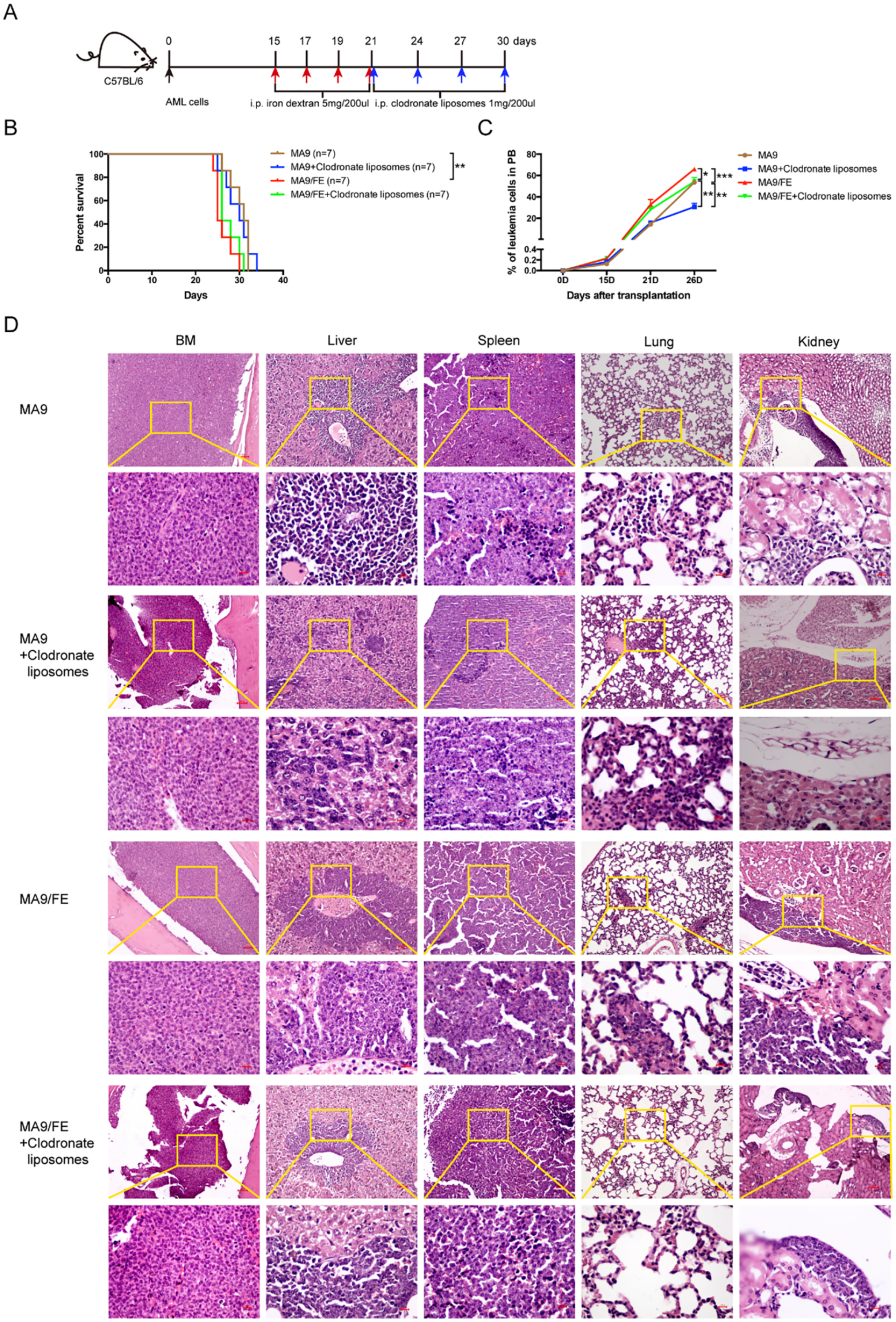
Depletion of LAMs hinders iron overload caused acceleration of AML progression. **(A)** Schematic of the experiment design is shown. **(B)** The mouse survival time was recorded, and Kaplan–Meier analysis was performed (n = 7). **(C)** PB leukemia cells were monitored at the indicated time points during the progression of leukemia. **(D)** Typical H&E-stained sections of BM, liver, spleen, lung, and kidney are shown. Scale bars (100 μm or 50 μm) are indicated. The results are from three independent experiments. Unpaired Student’s t test; *p < 0.05; **p < 0.01; ***p < 0.001.

Given the suppressive phagocytic function of LAMs and LAM-induced immune dysfunction in response to iron overload, we investigated whether *in vivo* LAM depletion alters the effects of iron overload on AML progression (**Figure 7A**). MA9/FE mice had a significantly shorter survival time than MA9 mice. Although it could not prolong the survival of either MA9 or MA9/FE mice (**Figure 7B**), LAM depletion by liposomal clodronate significantly decreased PB leukemia cell levels in both MA9 and MA9/FE mice on day 26 (**Figure 7C**). Tissue sections from leukemic mice were subjected to H&E staining showing that leukemia cell-infiltrating areas in tissues, including the BM, liver, and spleen, were significantly larger in MA9/FE mice than those in MA9 mice (**Figure 7D**). The infiltration of leukemia cells in the pulmonary veins was more severe in MA9/FE mice than that in MA9 mice. Additionally, a massive aggregation of leukemia cells was detected in the renal pelvis in MA9/FE mice. However, the depletion of LAMs decreased the infiltration of leukemia cells in tissues, including the BM, liver, spleen, lung and kidneys, in both MA9 and MA9/FE mice. Collectively, these findings indicate that iron overload shortens the survival time of AML mice, whereas the depletion of LAMs in iron-overload microenvironment hinders AML development.

## Discussion

In hematological malignancies, although iron overload in AML is not as common as in MDS, systemic iron overload is still a big clinical challenge as the results of ineffective hematopoiesis and disease-specific therapies. The increased iron level correlates with a poorer overall survival rate, a higher incidence of relapse and a suppressed autologous stem cell mobilization in AML patients (11, 12, 40). Nevertheless, the pathological significance of iron overload in AML has not received sufficient attention, and its pathological roles and mechanisms have not been elucidated. So, it is necessary to explore. The hematopoietic ecosystem comprises all cellular and non-cellular component of the hematopoietic system (41). Iron overload modifies the hematopoietic ecosystem to exert influence on normal or disease hematopoiesis. We previously dissected the direct effects of iron overload on leukemia cells (14). Here, we try to understand the indirect effects of iron overload on leukemia progression, *i.e.* the changes in other components of the hematopoietic ecosystem, specifically HSCs and LAMs. The results will give deeper understanding of the pathologic mechanisms of iron overload in leukemia.

HSCs not only maintain the decreased production of normal blood cells in leukemia, but also determine the effective recovery from the disease. Evidence has shown the progressively suppressed function of normal HSPCs in the impaired normal hematopoietic niche during leukemogenesis (17, 18, 28, 29). Furthermore, an intricate interplay between iron and HSCs has been revealed for HSC maintenance and fate decision (21, 22, 24, 42, 43). Both iron overload and iron deficiency disrupt HSC homeostasis, highlighting the delicate balance required for optimal hematopoietic function. Here, we report that iron overload did not further impact HSC maintenance in AML, which was distinct from those iron-induced changes in HSCs under steady state and stress conditions (21, 42, 43). This difference may due to the complexity of the disease-specific microenvironment. However, iron overload promoted the colony-forming capacity and further skewed myeloid differentiation of HSCs in AML. The myeloid-biased differentiation is one of the characteristics of senescent HSC (44, 45). HSC senescence affects the progression of leukemia, and is considered as a main factor resulting in the high incidence of AML in the elderly, which has important clinical implications. Intrinsic and extrinsic factors have been implicated in driving HSC senescence, such as DNA damage and mitochondrial damage, *etc.* (46, 47), which are also regulated by iron (48). Thus, iron overload may be involved in HSC senescence and play adverse roles in leukemia progression.

Different immune cells form the immunomicroenvironment in the hematopoietic ecosystem. They play sophisticated roles in leukemia (25, 28, 29, 49). Among them, macrophages attract much attention (50, 51). Although the M1 (classically activated macrophage) and M2 (alternatively activated macrophage) binary classification of macrophage activation phenotypes is well accepted (52), macrophage activation is more complex as they are signal-dependently activated into a continuum of states between the two extremes (31, 32). We previously found LAMs were educated by the leukemic microenvironment to exhibit unique phenotypes and functions (30–32). Recent studies suggested that iron metabolism was involved in regulating macrophage polarization (53), and induced tremendous plasticity of either M1- or M2- like phenotype under distinct conditions (33–38). These seemingly contradictory results highlight a context- dependent macrophage polarization. Here, we established a connection between iron metabolism and macrophage in AML. Iron overload correlates with increased M2 macrophages, which further correlate with a poor prognosis in iron overloaded AML patients. As the major iron storage protein, ferritin consists of the heavy and light chains, encoded by FTH1 and FTL genes. Using the expression level of FTH1 and FTL to reflect iron overload is not a specific indicator for iron overload in leukemia and has limitation. However, due to convenient sample collection and relatively low testing cost, ferritin is still considered as the preferred method for diagnosing iron overload in clinical practice. Dynamic monitoring the serum ferritin level in a cohort can more accurately determine iron overload status and give more solid conclusion. Nevertheless, analysis of transcriptional levels of FTH1 and FTL genes from public databases can also give clues for the correlation between macrophage activation and prognosis in iron overloaded AML patients. Using CD68 and CD163 to define macrophages and M2 macrophages in human is simple but has obvious limitations. Despite the fact that M2 macrophages have great heterogeneity and there is no well-accepted transcriptional profile to define M2 macrophages, using multiple genes or even whole genome transcriptional profile will improve the performance. The approaches like CIBERSORT-based immune de-convolution may give better results. However, due to the simplicity and convenience during the detection process at any time, using a typical marker to define macrophages is still a well-adopted method in the literature. Furthermore, we demonstrated a phenotypic shift, i.e. iron overload educated LAMs to exhibit more M2 characteristics. It’s well accepted that M1 or M1-like macrophages have anti-tumoral functions while M2 or M2-like macrophages are pro-tumoral (54). Therefore, overloaded iron may further educate LAMs to have more pro-leukemic effects.

Phagocytosis is an important functional characteristic of macrophages. It’s an effective strategy in cancer immunotherapy to potentiate macrophages for phagocytosis. We observed that iron overload decreased the phagocytic potential of LAMs, suggesting a decreased anti-leukemic effect. CD47/SIRPα axis is a major pathway in repressing the phagocytic function of macrophages (55, 56). CD47 was upregulated in iron-overload AML cells, which was consistent with the results in hMRP8bcr/abl × hMRP8bcl2 leukemic cells (57). By contrast, no difference was detected in the expression of SIRPα between MA9 and MA9/FE LAMs. Therefore, iron overload further inhibited the phagocytosis of LAMs against leukemia cells. These events may at least partly account for the accelerated AML progression mediated by iron overload.

Macrophages are an essential regulator of the intricate immune regulatory network in disease microenvironments. AML associated immune dysfunction is also mediated by T cells with impaired phenotypes (58–61), which is considered to be directly induced by malignant cells. However, we previously suggested the crosstalk between LAMs and T cells, and proved that LAMs directly regulated T cell activation (31). Here, we further demonstrated that iron overload promoted LAMs-mediated immunosuppression on T cells in several ways. On the one hand, the decreased CXCL11 level, a potent chemoattractant for T cells acting (62), expressed by LAMs in iron-overload mice, may contribute to fewer effector T cells in AML. On the other hand, iron overload induced the skewing of LAMs toward a tolerogenic phenotype with aberrant immune checkpoint expressions, which may contribute to T cell exhaustion leading to immune escape and progression acceleration in AML. Hence, due to the defective phenotype and suppressed functions of LAMs caused by iron overload, we postulated that LAM depletion might decrease the effects of iron overload on AML. Successfully, LAM depletion by liposomal clodronate attenuated iron overload-mediated acceleration of AML progression. In line with this result, macrophage depletion also delayed chronic lymphocytic leukemia development (63). Hence, our data emphasize that *in vivo* manipulation of macrophages has a pronounced impact on iron overloaded AML progression and suggest that AML should be an excellent candidate for cancer immunotherapy.

Taken together, we demonstrate that iron overload has a significant impact on normal HSCs and LAMs in the leukemic hematopoietic ecosystem, which contribute to the adverse roles of iron overload in AML progression. Therefore, we identify that the overloaded iron is a vital factor driving remodeling the BM microenvironment in AML. These findings provide new insights into the pathophysiologic mechanisms of iron overload in AML and broaden the knowledge of multifaceted roles of iron overload in malignancies.

## Data Availability

The datasets presented in this study can be found in online repositories. The names of the repository/repositories and accession number(s) can be found in the article/**Supplementary Material**.

## Supplementary material

Supplementary Figure 1 Iron overload validation in iron overloaded MLL-AF9-induced AML mouse model. Perl’s iron staining of the BM, liver, spleen, heart, lung, and kidney. Scale bars (100 μm) are indicated. The results are from three independent experiments.

Supplementary Figure 2 Representative morphological characteristics of the HSC colony types and gating strategy for donor-derived CD45.2^+^ cells in the PB and BM of recipients. **(A)** Typical BFU-E, CFU-GM, and CFU-GEMM colonies observed in this study are shown. **(B)** Representative FACS plots show the chimerism of donor-derived CD45.2^+^ in the PB of recipients during 16 weeks post-transplantation (n = 6). **(C)** Representative FACS plots show the chimerism of donor-derived CD45.2^+^ in the BM of recipients at 16 weeks post-transplantation (n = 6). **(D)** Representative FACS plots show the chimerism of donor-derived CD45.2^+^ HPC subpopulations (GMP: CD34^+^CD16/32^+^, CMP: CD34^+^ CD16/32^−^, MEP: CD34^-^CD16/32^-^, based on CD45.2^+^ Lin^-^Sca1^-^c-Kit^+^ population) in the BM of recipients at 16 weeks post-transplantation (n = 6). The results are from three independent experiments.

Supplementary Figure 3 Venn diagram of DEGs from different pairs of macrophages. Macrophages were sorted from the BM of MA9, MA9/FE mice and their respective normal counterparts (WT and WT/FE mice), and RNA-seq was performed. Venn diagram shows overlaps of the DEGs obtained from different pairs of macrophages.

Supplementary Figure 4 Typical FACS results of experiments testing the non-specific and specific phagocytic activity of macrophages. **(A)** Representative FACS plots show the non-specific phagocytic activity of macrophages. **(B)** Representative FACS plots show the lived AML cells cultured with or without macrophages. The results are from three independent experiments.

Supplementary Figure 5 Typical FACS results of immune checkpoint genes expressed on T cells cocultured with macrophages. Freshly sorted T cells from the spleen of WT mice were cocultured with macrophages from the BM of WT, WT/FE, MA9 or MA9/FE mice in 48-well plates for 48 hrs. T cells cultured without macrophages were set as the blank control. Expressions of PD-1, CD200R and TIM-3 in CD4^+^ **(A-C)** or CD8^+^ **(D-F)** T cells were detected by FACS. The results are from three independent experiments.

## References

[1] Möller HE Bossoni L Connor JR Crichton RR Does MD Ward RJ. Iron, myelin, and the brain: neuroimaging meets neurobiology. Trends Neurosci. (2019) 42:384–401. doi: 10.1016/j.tins.2019.03.009, PMID: 31047721

[2] Gao H Jin Z Bandyopadhyay G Wang G Zhang D Rocha KCE. Aberrant iron distribution via hepatocyte-stellate cell axis drives liver lipogenesis and fibrosis. Cell Metab. (2022) 34:1201–1213. doi: 10.1016/j.cmet.2022.07.006, PMID: 35921818 PMC9365100

[3] Rhee J-W Yi H Thomas D Lam CK Belbachir N Tian L. Modeling secondary iron overload cardiomyopathy with human induced pluripotent stem cell-derived cardiomyocytes. Cell Rep. (2020) 32:107886. doi: 10.1016/j.celrep.2020.107886, PMID: 32668256 PMC7553857

[4] Roemhild K von Maltzahn F Weiskirchen R Knüchel R von Stillfried S Lammers T. Iron metabolism: pathophysiology and pharmacology. Trends Pharmacol Sci. (2021) 42:640–56. doi: 10.1016/j.tips.2021.05.001, PMID: 34090703 PMC7611894

[5] Koleini N Shapiro JS Geier J Ardehali H. Ironing out mechanisms of iron homeostasis and disorders of iron deficiency. J Clin Invest. (2021) 131:e148671. doi: 10.1172/JCI148671, PMID: 34060484 PMC8159681

[6] Franke G-N Kubasch AS Cross M Vucinic V Platzbecker U. Iron overload and its impact on outcome of patients with hematological diseases. Mol aspects Med. (2020) 75:100868. doi: 10.1016/j.mam.2020.100868, PMID: 32620237

[7] Döhner H Weisdorf DJ Bloomfield CD. Acute myeloid leukemia. New Engl J Med. (2015) 373:1136–52. doi: 10.1056/NEJMra1406184, PMID: 26376137

[8] Newell LF Cook RJ. Advances in acute myeloid leukemia. BMJ. (2021) 375:n2026. doi: 10.1136/bmj.n2026, PMID: 34615640

[9] Aifantis I Raetz E Buonamici S. Molecular pathogenesis of T-cell leukaemia and lymphoma. Nat Rev Immunol. (2008) 8:380–90. doi: 10.1038/nri2304, PMID: 18421304

[10] Lyons RM Marek BJ Paley C Esposito J McNamara K Richards PD. Relation between chelation and clinical outcomes in lower-risk patients with myelodysplastic syndromes: Registry analysis at 5 years. Leukemia Res. (2017) 56:88–95. doi: 10.1016/j.leukres.2017.01.033, PMID: 28242540

[11] Wermke M Eckoldt J Götze KS Klein SA Bug G de Wreede LC. Enhanced labile plasma iron and outcome in acute myeloid leukaemia and myelodysplastic syndrome after allogeneic haemopoietic cell transplantation (ALLIVE): a prospective, multicentre, observational trial. Lancet Haematol. (2018) 5:e201–e10. doi: 10.1016/S2352-3026(18)30036-X, PMID: 29628397

[12] Bertoli S Paubelle E Bérard E Saland E Thomas X Tavitian S. Ferritin heavy/light chain (FTH1/FTL) expression, serum ferritin levels, and their functional as well as prognostic roles in acute myeloid leukemia. Eur J Haematol. (2019) 102:131–42. doi: 10.1111/ejh.13183, PMID: 30325535

[13] Alva LC Bacher U Seipel K Mansouri Taleghani B Mueller BU Novak U. Iron overload is correlated with impaired autologous stem cell mobilization and survival in acute myeloid leukemia. Transfusion. (2018) 58:2365–73. doi: 10.1111/trf.14895, PMID: 30203418

[14] Yang F Cui X Wang H Zhang D Luo S Li Y. Iron overload promotes the progression of MLL-AF9 induced acute myeloid leukemia by upregulation of FOS. Cancer letters. (2024) 583:216652. doi: 10.1016/j.canlet.2024.216652, PMID: 38242196

[15] Joshi SK Nechiporuk T Bottomly D Piehowski PD Reisz JA Pittsenbarger J. The AML microenvironment catalyzes a stepwise evolution to gilteritinib resistance. Cancer Cell. (2021) 39:999–1014. doi: 10.1016/j.ccell.2021.06.003, PMID: 34171263 PMC8686208

[16] Duarte D Hawkins ED Lo Celso C. The interplay of leukemia cells and the bone marrow microenvironment. Blood. (2018) 131:1507–11. doi: 10.1182/blood-2017-12-784132, PMID: 29487069

[17] Cheng H Hao S Liu Y Pang Y Ma S Dong F. Leukemic marrow infiltration reveals a novel role for Egr3 as a potent inhibitor of normal hematopoietic stem cell proliferation. Blood. (2015) 126:1302–13. doi: 10.1182/blood-2015-01-623645, PMID: 26186938 PMC4574014

[18] Hu X Shen H Tian C Yu H Zheng G XuFeng R. Kinetics of normal hematopoietic stem and progenitor cells in a Notch1-induced leukemia model. Blood. (2009) 114:3783–92. doi: 10.1182/blood-2009-06-227843, PMID: 19652197 PMC2773494

[19] Vago L Gojo I. Immune escape and immunotherapy of acute myeloid leukemia. J Clin Invest. (2020) 130:1552–64. doi: 10.1172/JCI129204, PMID: 32235097 PMC7108895

[20] Tang X Wang Z Wang J Cui S Xu R Wang Y. Functions and regulatory mechanisms of resting hematopoietic stem cells: a promising targeted therapeutic strategy. Stem Cell Res Ther. (2023) 14:73. doi: 10.1186/s13287-023-03316-5, PMID: 37038215 PMC10088186

[21] Zhang D Gao X Li H Borger DK Wei Q Yang E. The microbiota regulates hematopoietic stem cell fate decisions by controlling iron availability in bone marrow. Cell Stem Cell. (2022) 29:232–247. doi: 10.1016/j.stem.2021.12.009, PMID: 35065706 PMC8818037

[22] Wang S He X Wu Q Jiang L Chen L Yu Y. Transferrin receptor 1-mediated iron uptake plays an essential role in hematopoiesis. Haematologica. (2020) 105:2071–82. doi: 10.3324/haematol.2019.224899, PMID: 31601687 PMC7395265

[23] Talkhoncheh MS Baudet A Ek F Subramaniam A Kao Y-R Miharada N. Ciclopirox ethanolamine preserves the immature state of human HSCs by mediating intracellular iron content. Blood Adv. (2023) 7:7407–17. doi: 10.1182/bloodadvances.2023009844, PMID: 37487020 PMC10758717

[24] Yi W Zhang J Huang Y Zhan Q Zou M Cheng X. Ferritin-mediated mitochondrial iron homeostasis is essential for the survival of hematopoietic stem cells and leukemic stem cells. Leukemia. (2024) 38:1003–18. doi: 10.1038/s41375-024-02169-y, PMID: 38402368

[25] Hu T Cheng B Matsunaga A Zhang T Lu X Fang H. Single-cell analysis defines highly specific leukemia-induced neutrophils and links MMP8 expression to recruitment of tumor associated neutrophils during FGFR1 driven leukemogenesis. Exp Hematol Oncol. (2024) 13:49. doi: 10.1186/s40164-024-00514-6, PMID: 38730491 PMC11084112

[26] Li W Wang F Guo R Bian Z Song Y. Targeting macrophages in hematological Malignancies: recent advances and future directions. J Hematol Oncol. (2022) 15:110. doi: 10.1186/s13045-022-01328-x, PMID: 35978372 PMC9387027

[27] Crinier A Dumas P-Y Escalière B Piperoglou C Gil L Villacreces A. Single-cell profiling reveals the trajectories of natural killer cell differentiation in bone marrow and a stress signature induced by acute myeloid leukemia. Cell Mol Immunol. (2021) 18:1290–304. doi: 10.1038/s41423-020-00574-8, PMID: 33239726 PMC8093261

[28] Yang F Wang R Feng W Chen C Yang X Wang L. Characteristics of NK cells from leukemic microenvironment in MLL-AF9 induced acute myeloid leukemia. Mol Immunol. (2018) 93:68–78. doi: 10.1016/j.molimm.2017.11.003, PMID: 29154208

[29] Wang R Feng W Wang H Wang L Yang X Yang F. Blocking migration of regulatory T cells to leukemic hematopoietic microenvironment delays disease progression in mouse leukemia model. Cancer letters. (2020) 469:151–61. doi: 10.1016/j.canlet.2019.10.032, PMID: 31669202

[30] Chen S-Y Yang X Feng W-L Liao J-F Wang L-N Feng L. Organ-specific microenvironment modifies diverse functional and phenotypic characteristics of leukemia-associated macrophages in mouse T cell acute lymphoblastic leukemia. J Immunol (Baltimore Md: 1950). (2015) 194:2919–29. doi: 10.4049/jimmunol.1400451, PMID: 25662994

[31] Yang X Feng W Wang R Yang F Wang L Chen S. Repolarizing heterogeneous leukemia-associated macrophages with more M1 characteristics eliminates their pro-leukemic effects. Oncoimmunology. (2018) 7:e1412910. doi: 10.1080/2162402X.2017.1412910, PMID: 29632729 PMC5889280

[32] Yang F Feng W Wang H Wang L Liu X Wang R. Monocyte-derived leukemia-associated macrophages facilitate extramedullary distribution of T-cell acute lymphoblastic leukemia cells. Cancer Res. (2020) 80:3677–91. doi: 10.1158/0008-5472.CAN-20-0034, PMID: 32651260

[33] Jais A Einwallner E Sharif O Gossens K Lu TT Soyal SM. Heme oxygenase-1 drives metaflammation and insulin resistance in mouse and man. Cell. (2014) 158:25–40. doi: 10.1016/j.cell.2014.04.043, PMID: 24995976 PMC5749244

[34] Handa P Thomas S Morgan-Stevenson V Maliken BD Gochanour E Boukhar S. Iron alters macrophage polarization status and leads to steatohepatitis and fibrogenesis. J leukocyte Biol. (2019) 105:1015–26. doi: 10.1002/JLB.3A0318-108R, PMID: 30835899

[35] Joffin N Gliniak CM Funcke J-B Paschoal VA Crewe C Chen S. Adipose tissue macrophages exert systemic metabolic control by manipulating local iron concentrations. Nat Metab. (2022) 4:1474–94. doi: 10.1038/s42255-022-00664-z, PMID: 36329217 PMC11750126

[36] Kao J-K Wang S-C Ho L-W Huang S-W Lee C-H Lee M-S. M2-like polarization of THP-1 monocyte-derived macrophages under chronic iron overload. Ann hematol. (2020) 99:431–41. doi: 10.1007/s00277-020-03916-8, PMID: 32006153

[37] Sun J-L Zhang N-P Xu R-C Zhang G-C Liu Z-Y Abuduwaili W. Tumor cell-imposed iron restriction drives immunosuppressive polarization of tumor-associated macrophages. J Transl Med. (2021) 19:347. doi: 10.1186/s12967-021-03034-7, PMID: 34389031 PMC8361643

[38] Wilkinson HN Roberts ER Stafford AR Banyard KL Matteucci P Mace KA. Tissue iron promotes wound repair via M2 macrophage polarization and the chemokine (C-C motif) ligands 17 and 22. Am J pathology. (2019) 189:2196–208. doi: 10.1016/j.ajpath.2019.07.015, PMID: 31465751 PMC12179499

[39] Zhang D Cui X Li Y Wang R Wang H Dai Y. Sox13 and M2-like leukemia-associated macrophages contribute to endogenous IL-34 caused accelerated progression of acute myeloid leukemia. Cell Death Dis. (2023) 14:308. doi: 10.1038/s41419-023-05822-z, PMID: 37149693 PMC10164149

[40] Weber S Parmon A Kurrle N Schnütgen F Serve H. The clinical significance of iron overload and iron metabolism in myelodysplastic syndrome and acute myeloid leukemia. Front Immunol. (2020) 11:627662. doi: 10.3389/fimmu.2020.627662, PMID: 33679722 PMC7933218

[41] Cheng H. Deciphering the hematopoietic cell ecosystem. Blood Sci. (2024) 6:e00211. doi: 10.1097/BS9.0000000000000211, PMID: 39620203 PMC11608643

[42] Tanaka H Espinoza JL Fujiwara R Rai S Morita Y Ashida T. Excessive reactive iron impairs hematopoiesis by affecting both immature hematopoietic cells and stromal cells. Cells. (2019) 8:226. doi: 10.3390/cells8030226, PMID: 30857202 PMC6468739

[43] Lu W Zhao M Rajbhandary S Xie F Chai X Mu J. Free iron catalyzes oxidative damage to hematopoietic cells/mesenchymal stem cells *in vitro* and suppresses hematopoiesis in iron overload patients. Eur J Haematol. (2013) 91:249–61. doi: 10.1111/ejh.12159, PMID: 23772810

[44] Zeng X Li X Li X Wei C Shi C Hu K. Fecal microbiota transplantation from young mice rejuvenates aged hematopoietic stem cells by suppressing inflammation. Blood. (2023) 141:1691–707. doi: 10.1182/blood.2022017514, PMID: 36638348 PMC10646769

[45] Abdelfattah A Hughes-Davies A Clayfield L Menendez-Gonzalez JB Almotiri A Alotaibi B. Gata2 haploinsufficiency promotes proliferation and functional decline of hematopoietic stem cells with myeloid bias during aging. Blood Adv. (2021) 5:4285–90. doi: 10.1182/bloodadvances.2021004726, PMID: 34496012 PMC8945642

[46] Wu L Lin Q Chatla S Amarachintha S Wilson AF Atale N. LepR+ niche cell-derived AREG compromises hematopoietic stem cell maintenance under conditions of DNA repair deficiency and aging. Blood. (2023) 142:1529–42. doi: 10.1182/blood.2022018212, PMID: 37584437 PMC10656728

[47] Mansell E Sigurdsson V Deltcheva E Brown J James C Miharada K. Mitochondrial potentiation ameliorates age-related heterogeneity in hematopoietic stem cell function. Cell Stem Cell. (2021) 28:241–256. doi: 10.1016/j.stem.2020.09.018, PMID: 33086034

[48] Zhang Q Chen C Zou X Wu W Di Y Li N. Iron promotes ovarian cancer Malignancy and advances platinum resistance by enhancing DNA repair via FTH1/FTL/POLQ/RAD51 axis. Cell Death Dis. (2024) 15:329. doi: 10.1038/s41419-024-06688-5, PMID: 38740757 PMC11091064

[49] Moore JA Mistry JJ Hellmich C Horton RH Wojtowicz EE Jibril A. LC3-associated phagocytosis in bone marrow macrophages suppresses acute myeloid leukemia progression through STING activation. J Clin Invest. (2022) 132:e153157. doi: 10.1172/JCI153157, PMID: 34990402 PMC8884913

[50] Zhang X Li S Malik I Do MH Ji L Chou C. Reprogramming tumour-associated macrophages to outcompete cancer cells. Nature. (2023) 619:616–23. doi: 10.1038/s41586-023-06256-5, PMID: 37380769 PMC10719927

[51] Xia Y Rao L Yao H Wang Z Ning P Chen X. Engineering macrophages for cancer immunotherapy and drug delivery. Adv Mater. (2020) 32:e2002054. doi: 10.1002/adma.202002054, PMID: 32856350

[52] Van Overmeire E Laoui D Keirsse J Van Ginderachter JA Sarukhan A. Mechanisms driving macrophage diversity and specialization in distinct tumor microenvironments and parallelisms with other tissues. Front Immunol. (2014) 5:127. doi: 10.3389/fimmu.2014.00127, PMID: 24723924 PMC3972476

[53] Soares MP Hamza I. Macrophages and iron metabolism. Immunity. (2016) 44:492–504. doi: 10.1016/j.immuni.2016.02.016, PMID: 26982356 PMC4794998

[54] Wang J Mi S Ding M Li X Yuan S. Metabolism and polarization regulation of macrophages in the tumor microenvironment. Cancer letters. (2022) 543:215766. doi: 10.1016/j.canlet.2022.215766, PMID: 35690285

[55] Morrissey MA Kern N Vale RD. CD47 ligation repositions the inhibitory receptor SIRPA to suppress integrin activation and phagocytosis. Immunity. (2020) 53:290–302. doi: 10.1016/j.immuni.2020.07.008, PMID: 32768386 PMC7453839

[56] McCracken MN Cha AC Weissman IL. Molecular pathways: activating T cells after cancer cell phagocytosis from blockade of CD47 “Don’t eat me” Signals. Clin Cancer Res. (2015) 21:3597–601. doi: 10.1158/1078-0432.CCR-14-2520, PMID: 26116271 PMC4621226

[57] Jaiswal S Jamieson CHM Pang WW Park CY Chao MP Majeti R. CD47 is upregulated on circulating hematopoietic stem cells and leukemia cells to avoid phagocytosis. Cell. (2009) 138:271–85. doi: 10.1016/j.cell.2009.05.046, PMID: 19632178 PMC2775564

[58] Kasakovski D Xu L Li Y. T cell senescence and CAR-T cell exhaustion in hematological Malignancies. J Hematol Oncol. (2018) 11:91. doi: 10.1186/s13045-018-0629-x, PMID: 29973238 PMC6032767

[59] Liu L Cheng X Yang H Lian S Jiang Y Liang J. BCL-2 expression promotes immunosuppression in chronic lymphocytic leukemia by enhancing regulatory T cell differentiation and cytotoxic T cell exhaustion. Mol Cancer. (2022) 21:59. doi: 10.1186/s12943-022-01516-w, PMID: 35193595 PMC8862474

[60] Tracy SI Venkatesh H Hekim C Heltemes-Harris LM Knutson TP Bachanova V. Combining nilotinib and PD-L1 blockade reverses CD4+ T-cell dysfunction and prevents relapse in acute B-cell leukemia. Blood. (2022) 140:335–48. doi: 10.1182/blood.2021015341, PMID: 35275990 PMC9335501

[61] Zhou Y-J Li G Wang J Liu M Wang Z Song Y. PD-L1: expression regulation. Blood Sci. (2023) 5:77–91. doi: 10.1097/BS9.0000000000000149, PMID: 37228770 PMC10205351

[62] de Masson A Darbord D Dobos G Boisson M Roelens M Ram-Wolff C. Macrophage-derived CXCL9 and CXCL11, T-cell skin homing, and disease control in mogamulizumab-treated CTCL patients. Blood. (2022) 139:1820–32. doi: 10.1182/blood.2021013341, PMID: 34905599

[63] Hanna BS McClanahan F Yazdanparast H Zaborsky N Kalter V Rößner PM. Depletion of CLL-associated patrolling monocytes and macrophages controls disease development and repairs immune dysfunction *in vivo* . Leukemia. (2016) 30:570–9. doi: 10.1038/leu.2015.305, PMID: 26522085

