# Supplementary figures and images for: Iron overload promotes myeloid differentiation of normal hematopoietic stem cells and educates macrophage mediated immunosuppression in acute myeloid leukemia
Source: Front Immunol. 2025 Aug 13;16:1626888. doi: 10.3389/fimmu.2025.1626888 (PMC12380764; doi:10.3389/fimmu.2025.1626888)

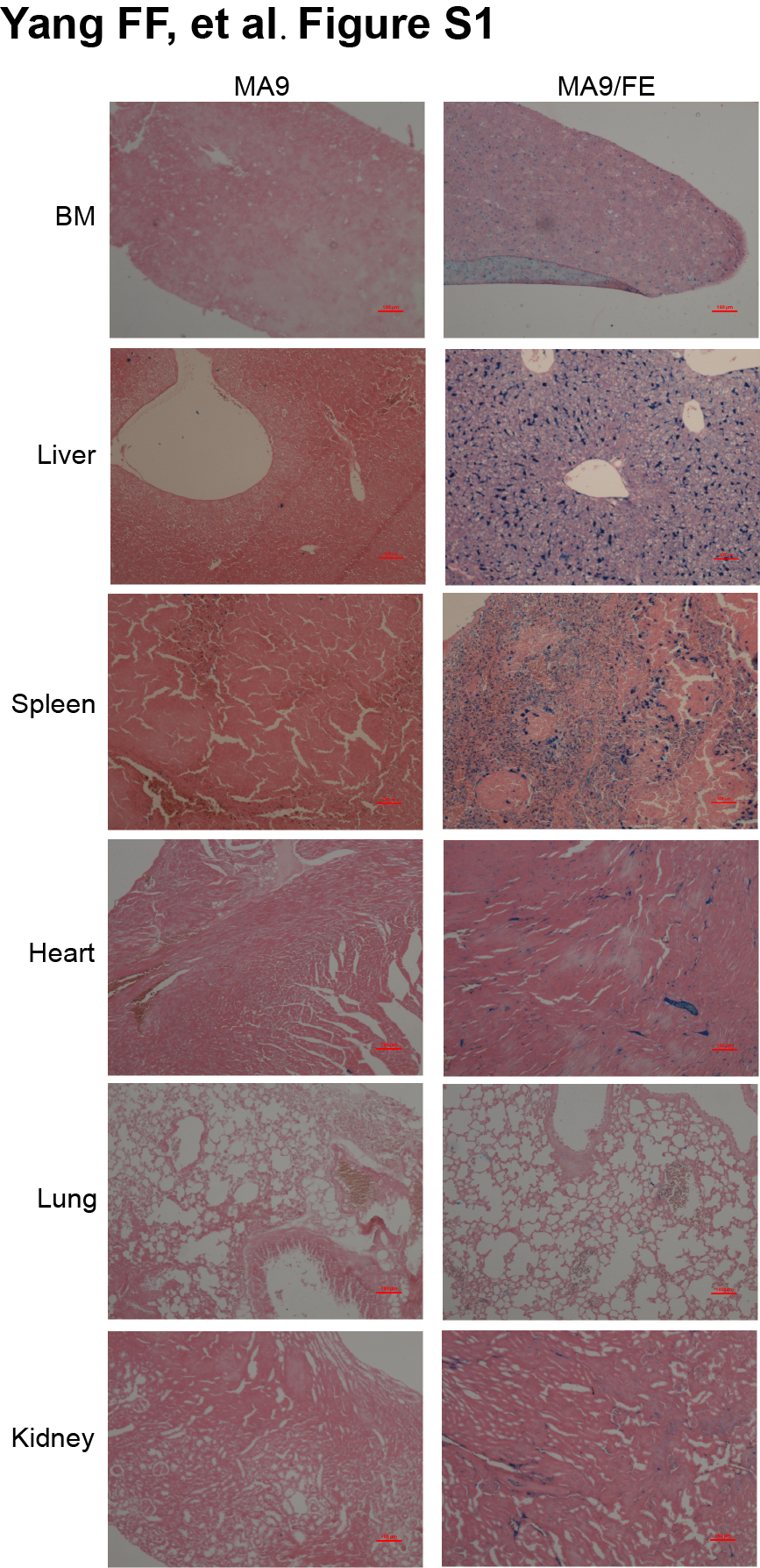

Supplement: Supplementary Figure 1 — Iron overload validation in iron overloaded MLL-AF9-induced AML mouse model. Perl’s iron staining of the BM, liver, spleen, heart, lung, and kidney. Scale bars (100 μm) are indicated. The results are from three independent experiments. [file Image1.png]

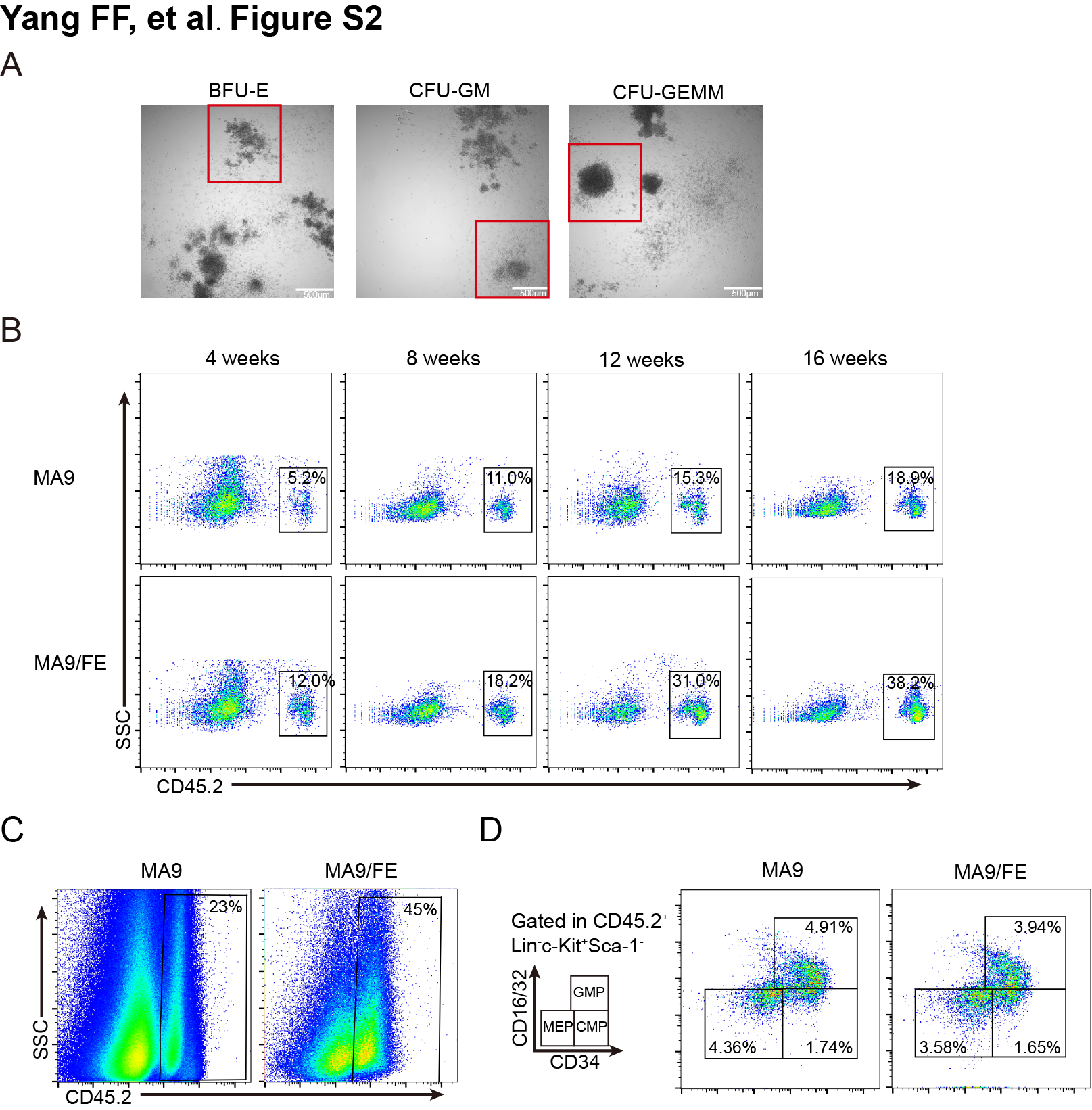

Supplement: Supplementary Figure 2 — Representative morphological characteristics of the HSC colony types and gating strategy for donor-derived CD45.2+ cells in the PB and BM of recipients. (A) Typical BFU-E, CFU-GM, and CFU-GEMM colonies observed in this study are shown. (B) Representative FACS plots show the chimerism of donor-derived CD45.2+ in the PB of recipients during 16 weeks post-transplantation (n = 6). (C) Representative FACS plots show the chimerism of donor-derived CD45.2+ in the BM of recipients at 16 weeks post-transplantation (n = 6). (D) Representative FACS plots show the chimerism of donor-derived CD45.2+ HPC subpopulations (GMP: CD34+CD16/32+, CMP: CD34+ CD16/32−, MEP: CD34-CD16/32-, based on CD45.2+ Lin-Sca1-c-Kit+ population) in the BM of recipients at 16 weeks post-transplantation (n = 6). The results are from three independent experiments. [file Image2.png]

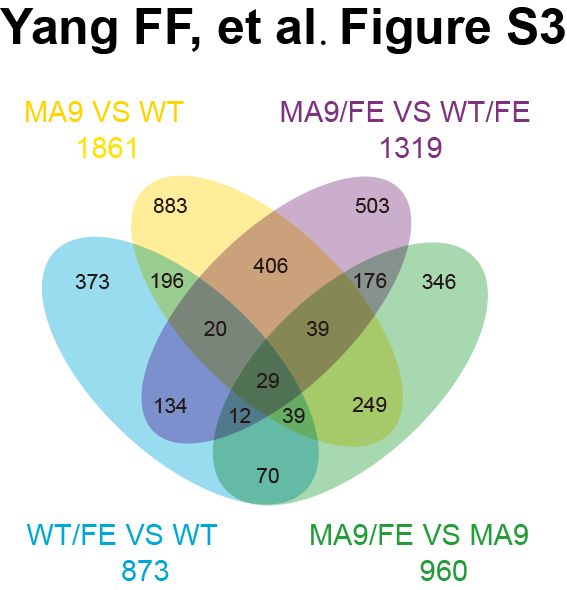

Supplement: Supplementary Figure 3 — Venn diagram of DEGs from different pairs of macrophages. Macrophages were sorted from the BM of MA9, MA9/FE mice and their respective normal counterparts (WT and WT/FE mice), and RNA-seq was performed. Venn diagram shows overlaps of the DEGs obtained from different pairs of macrophages. [file Image3.png]

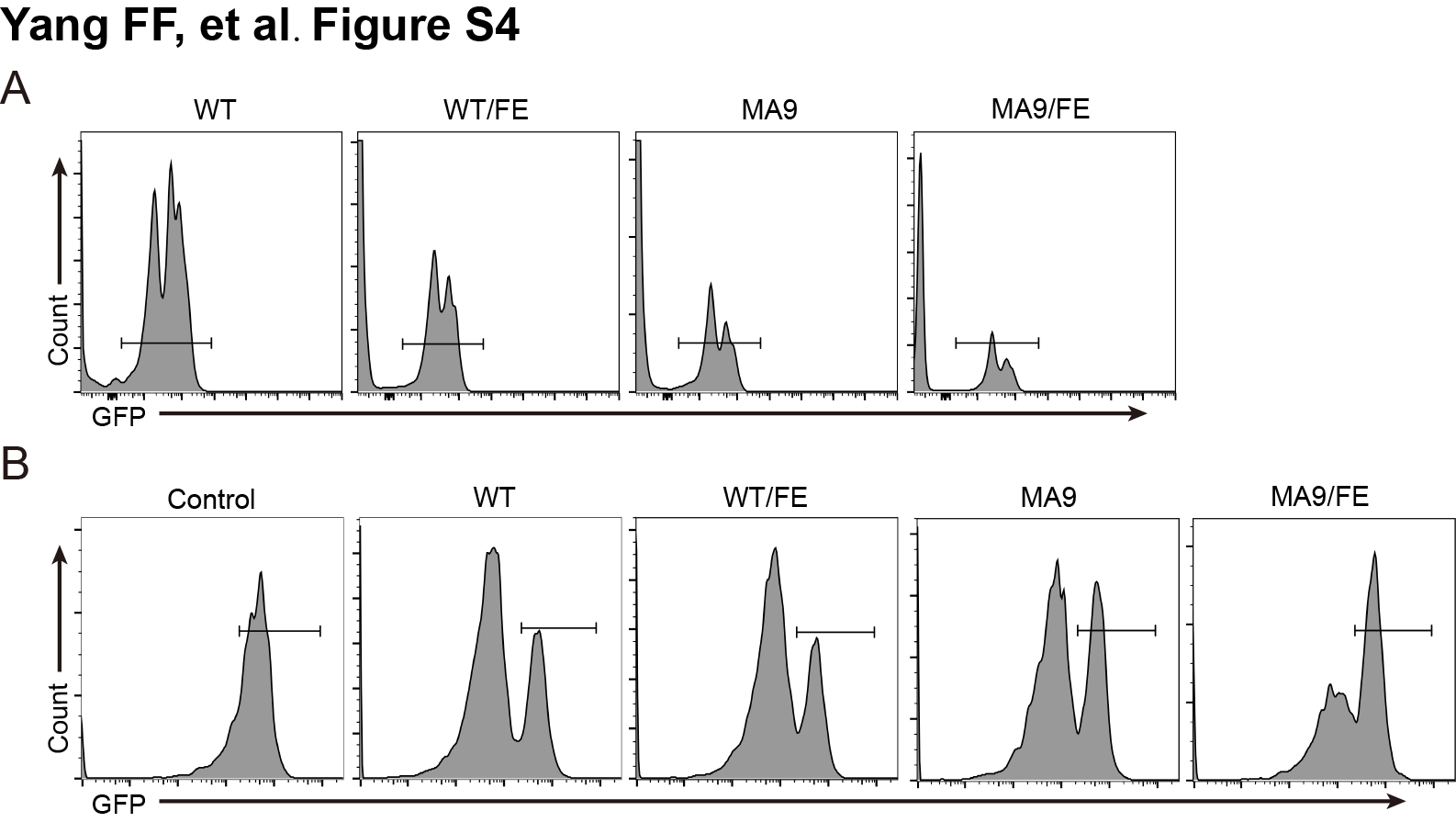

Supplement: Supplementary Figure 4 — Typical FACS results of experiments testing the non-specific and specific phagocytic activity of macrophages. (A) Representative FACS plots show the non-specific phagocytic activity of macrophages. (B) Representative FACS plots show the lived AML cells cultured with or without macrophages. The results are from three independent experiments. [file Image4.png]

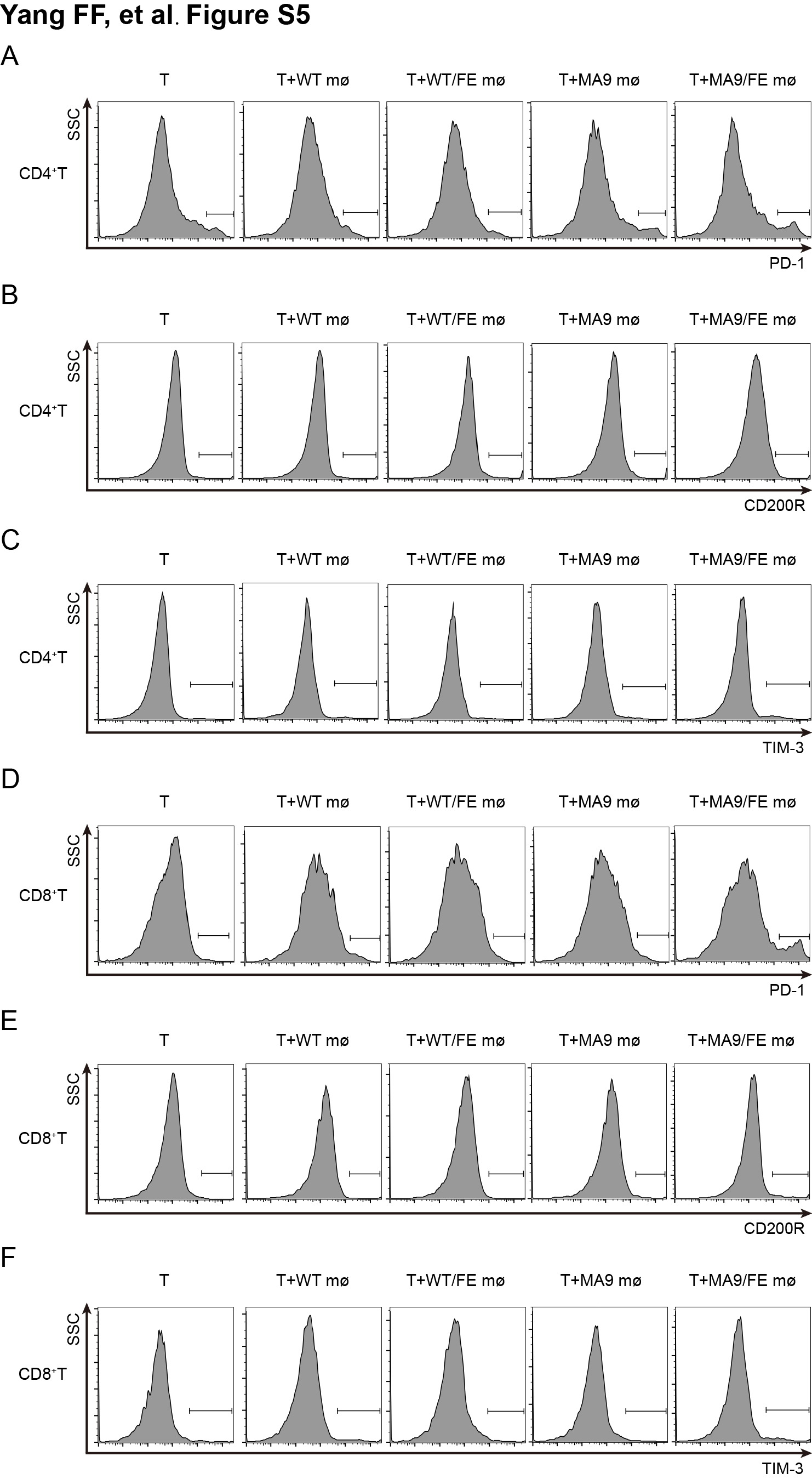

Supplement: Supplementary Figure 5 — Typical FACS results of immune checkpoint genes expressed on T cells cocultured with macrophages. Freshly sorted T cells from the spleen of WT mice were cocultured with macrophages from the BM of WT, WT/FE, MA9 or MA9/FE mice in 48-well plates for 48 hrs. T cells cultured without macrophages were set as the blank control. Expressions of PD-1, CD200R and TIM-3 in CD4+ (A-C) or CD8+ (D-F) T cells were detected by FACS. The results are from three independent experiments. [file Image5.png]
