# Supplementary material for: Iron overload promotes myeloid differentiation of normal hematopoietic stem cells and educates macrophage mediated immunosuppression in acute myeloid leukemia
Source: Front Immunol. 2025 Aug 13;16:1626888. doi: 10.3389/fimmu.2025.1626888 (PMC12380764; doi:10.3389/fimmu.2025.1626888)
Supplement: Supplementary file 6 [file Table1.docx]

**Supplementary Table 1. Website to download detailed characteristics of leukemia patients from the public database.**

| **Database** | **Website** | **Patient Characteristics Provided** |
| --- | --- | --- |
| TCGA | https://portal.gdc.cancer.gov/repository?f  acetTab=cases&filters=%7B%22op%22 %3A%22and%22%2C%22content%22% 3A%5B%7B%22op%22%3A%22in%22 %2C%22content%22%3A%7B%22field %22%3A%22cases.primary_site%22%2 C%22value%22%3A%5B%22hematopoi etic%20and%20reticuloendothelial%20s ystems%22%5D%7D%7D%5D%7D&se archTableTab=cases | Gender, age, race, bone marrow blast percentage, PB blast percentage, WBC, AML in skin percentage, subclones, cytogenetics, genomic rearrangement, cytogenetic code, histological subtype, risk (Cyto), risk (Molecular), structural variants from WGS, DFS months, DFS status, OS months, OS status, induction, type of transplant (sib Allo, MUD, Auto, etc) , dark zone stat at transplant, disease type, cancer type detailed |
