# Supplementary material for: Iron overload promotes myeloid differentiation of normal hematopoietic stem cells and educates macrophage mediated immunosuppression in acute myeloid leukemia
Source: Front Immunol. 2025 Aug 13;16:1626888. doi: 10.3389/fimmu.2025.1626888 (PMC12380764; doi:10.3389/fimmu.2025.1626888)
Supplement: Supplementary file 7 [file Table2.doc]

**Supplementary Table 2. Primers used in real-time RT-PCR.**

| **Gene** | **Forward (5’-3’)** | **Reverse (5’-3’)** |
| --- | --- | --- |
| GAPDH | CACTTGAAGGGTGGAGC | GGGCTAAGCAGTTGGTG |
| IL-1β | TGCCACCTTTTGACAGTGAT | TGTCCTCATCCTGGAAGGTC |
| IL-6 | CCGCTATGAAGTTCCTCTCTGC | ATCCTCTGTGAAGTCTCCTCTCC |
| IL-10 | CCAGAGCCACATGCTCCTA | AGGGGAGAAATCGATGACAG |
| IL-12β | ATGTGGAATGGCGTCTCTGTCT | TGGGCGGGTCTGGTTTGA |
| iNOS | CAGCGGAGTGACGGCAAAC | AGACCAGAGGCAGCACATCAA |
| TNF-α | CAACCAGCTCTGGGAATCTG | GGCACCACTAGTTGGTTGTCTTTG |
| MRC1 | CCTGAACAGCAACTTGACCA | GCAATGGCCATAGAAAGGAA |
| MMP9 | TGAGTCCGGCAGACAATCCT | CCCTGGATCTCAGCAATAGCA |
| AGR1 | CAACCAGCTCTGGGAATCTG | AATCGGCCTTTTCTTCCTTC |
| CXCL11 | TGAGTCCGGCAGACAATCCT | CCCTGGATCTCAGCAATAGCA |
| CCL17 | TGCTTCTGGGGACTTTTCTG | AATCGGCCTTTTCTTCCTTC |
| CSF1 | TCACAACCTCATCCTTCTGCG | GACCCAGTTAGTGCCCAGTGA |
